# Target prediction and validation of microRNAs expressed from FSHR and aromatase genes in human ovarian granulosa cells

**DOI:** 10.1038/s41598-020-59186-x

**Published:** 2020-02-10

**Authors:** Ilmatar Rooda, Kati Hensen, Birgitta Kaselt, Sergo Kasvandik, Martin Pook, Ants Kurg, Andres Salumets, Agne Velthut-Meikas

**Affiliations:** 1 Department of Chemistry and Biotechnology, Tallinn University of Technology, Tallinn, Estonia; 2 Competence Centre on Health Technologies, Tartu, Estonia; 3 Institute of Molecular and Cell Biology, University of Tartu, Tartu, Estonia; 4 Proteomics Core Facility, Institute of Technology, University of Tartu, Tartu, Estonia; 5 Institute of Clinical Medicine, Department of Obstetrics and Gynecology, University of Tartu, Tartu, Estonia; 6 Institute of Biomedicine and Translational Medicine, Department of Biomedicine, University of Tartu, Tartu, Estonia; 7 Department of Obstetrics and Gynecology, University of Helsinki and Helsinki University Hospital, Helsinki, Finland

**Keywords:** miRNAs, Transcriptomics, Ovary

## Abstract

MicroRNAs (miRNAs) are known post-transcriptional regulators of various biological processes including ovarian follicle development. We have previously identified miRNAs from human pre-ovulatory ovarian granulosa cells that are expressed from the intronic regions of two key genes in normal follicular development: FSH receptor (*FSHR*) and *CYP19A1*, the latter encoding the aromatase enzyme. The present study aims to identify the target genes regulated by these miRNAs: hsa-miR-548ba and hsa-miR-7973, respectively. The miRNAs of interest were transfected into KGN cell line and the gene expression changes were analyzed by Affymetrix microarray. Potential miRNA-regulated genes were further filtered by bioinformatic target prediction algorithms and validated for direct miRNA:mRNA binding by luciferase reporter assay. *LIFR*, *PTEN*, *NEO1* and *SP110* were confirmed as targets for hsa-miR-548ba. Hsa-miR-7973 target genes *ADAM19*, *PXDN* and *FMNL3* also passed all verification steps. Additionally, the expression pattern of the miRNAs was studied in human primary cumulus granulosa cell culture in relation to the expression of their host genes and FSH stimulation. Based on our findings we propose the involvement of hsa-miR-548ba in the regulation of follicle growth and activation via LIFR and PTEN. Hsa-miR-7973 may be implicated in the modulation of extracellular matrix and cell-cell interactions by regulating the expression of its identified targets.

## Introduction

Granulosa cell functions are essential in follicular development, maturation, and atresia. Granulosa cells support oocyte growth via continuous bidirectional communication to ensure oocyte quality and developmental competence. Due to the close communication cumulus (CGC) and mural granulosa cells (MGC) may reflect the characteristics of the oocyte and an understanding of the patterns of expression and functions of miRNAs in those cells may lead to a better understanding of follicle maturation and its dysfunction^1,2^.

MicroRNAs (miRNAs) are short non-coding RNA molecules approximately 22 nucleotides in length^3^. MicroRNAs bind to their target mRNA 3′ untranslated region (3′UTR) at sites complementary to the miRNA 5′ seed region. MicroRNA biding to the target gene regulates gene expression by destabilizing the mRNA and/or inhibiting its translation^4^. In a few cases, an increase in gene expression has also been observed^5^.

MicroRNAs have key role in the post-transcriptional regulation of various important biological processes, including cell proliferation, differentiation, apoptosis, and hormone biosynthesis and secretion^6^. Functional ovary requires precise coordination of follicle recruitment, selection and ovulation processes. Development of ovarian follicles is a complex process including oocyte maturation, granulosa cell proliferation and differentiation. MicroRNAs expressed in the ovary have regulative roles in ovarian follicle development and the expression of miRNAs in the ovary varies within the specific cell type and function^7^. The overall role of miRNAs in ovarian functions has been extensively studied in the mouse model as well as in humans. *Dicer1* conditional knock-out mice without the ability to process mature miRNAs in anti-Müllerian hormone expressing cells, including follicular granulosa cells, demonstrate abnormalities in ovulation, early embryonic development, and estrous cycles^8^. miRNA expression levels in CGC is altered in women diagnosed with polycystic ovary syndrome^9^. Additionally, there is a difference in the miRNA profile of CGC related to the meiotic maturation stage of the corresponding oocyte^10^. Therefore, granulosa cell miRNAs may serve as potential biological markers to increase the efficiency of assisted reproductive technologies by providing non-invasive means to assess oocyte quality and embryo survival potential^1^.

miRNAs hsa-miR-548ba and hsa-miR-7973 were previously identified by deep sequencing of MGC and CGC populations isolated from women undergoing controlled ovarian stimulation and *in vitro* fertilization. Both miRNAs are of intronic origin: hsa-miR-548ba gene resides in the follicle stimulating hormone receptor (*FSHR*) gene and hsa-miR-7973 is located in the intron of the *CYP19A1* gene^11^. The regulatory mechanisms and target genes for those two miRNAs are currently not known.

Follicle stimulating hormone (FSH) activates time-related changes in granulosa cell gene expression by binding to FSHR promoting proliferation, differentiation, antrum formation, and oocyte maturation. Moreover, FSH stimulates aromatase expression and estrogens production^12,13^. Estrogens are produced by aromatization of androgens by aromatase enzyme encoded from *CYP19A1* gene^14^. Both FSHR and aromatase are crucial for follicle development and maturation^13^.

The genomic locations of hsa-miR-548ba and hsa-miR-7973 in *FSHR* and aromatase genes, respectively, refers to potentially important regulatory roles of these miRNAs in follicle development and function. The primary aim of the current study was to identify the target genes of hsa-miR-548ba and hsa-miR-7973 in human granulosa cells by using granulosa KGN cell line as a model^15^. Secondly, the dependency of endogenous miRNA expression on their host genes and on FSH stimulation is investigated in primary human granulosa cells.

## Results

**Figure 1:**
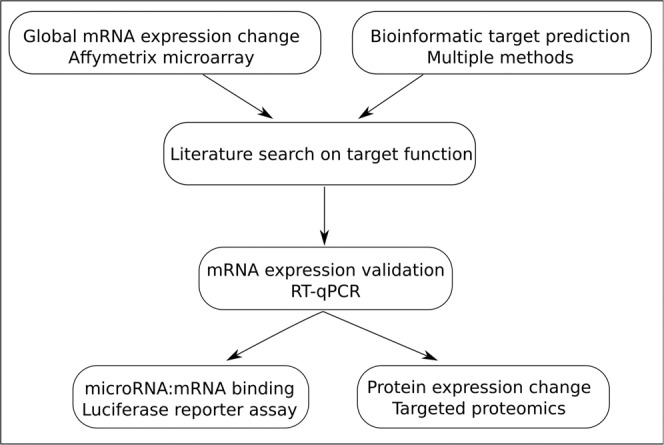
The rationale and methods used to identify and validate miRNA targets. Each arrow represents a filtering step, the conditions of which are specified in the text.

Multiple methods and selection criteria were used to identify and narrow down the potential targets of hsa-miR-548ba and hsa-miR-7973. The methodological rationale for filtering the potential targets is depicted in Fig. 1.

### Global gene expression changes upon transient expression of hsa-miR-548ba and hsa-miR-7973 in KGN cells

The first aim of the current study was to evaluate the effect of miRNA transfection on the global gene expression change in human granulosa cell line KGN. In non-transfected KGN cells the expression levels of hsa-miR-548ba and hsa-miR-7973 barely reached the detection limit (Supplementary Fig. 1). After optimization experiments (data not shown), the transfection of 12.5 nM miRNA mimic lead to considerably higher expression levels in comparison to primary granulosa cells (Supplementary Fig. 1). However, such level of over-expression did not influence cell viability or proliferation rate (Supplementary Fig. 2).

Genome-wide gene expression changes upon miRNA transfection were studied on Affymetrix GeneChip Human Gene 2.0 ST Array. The results demonstrated that upon hsa-miR-548ba transfection the expression level of 1,474 and upon hsa-miR-7973 the expression level of 1,552 genes changed with statistical significance (adjusted p-value < 0.01, Supplementary Table IIA,C). From those genes 1,015 were regulated by both miRNAs, 459 genes only by hsa-miR-548ba and 537 by hsa-miR-7973. Gene expression changes were calculated in comparison to the control samples transfected with miRNA cel-miR-39-3p that presumably has no target sequences in human cells.

**Figure 2:**
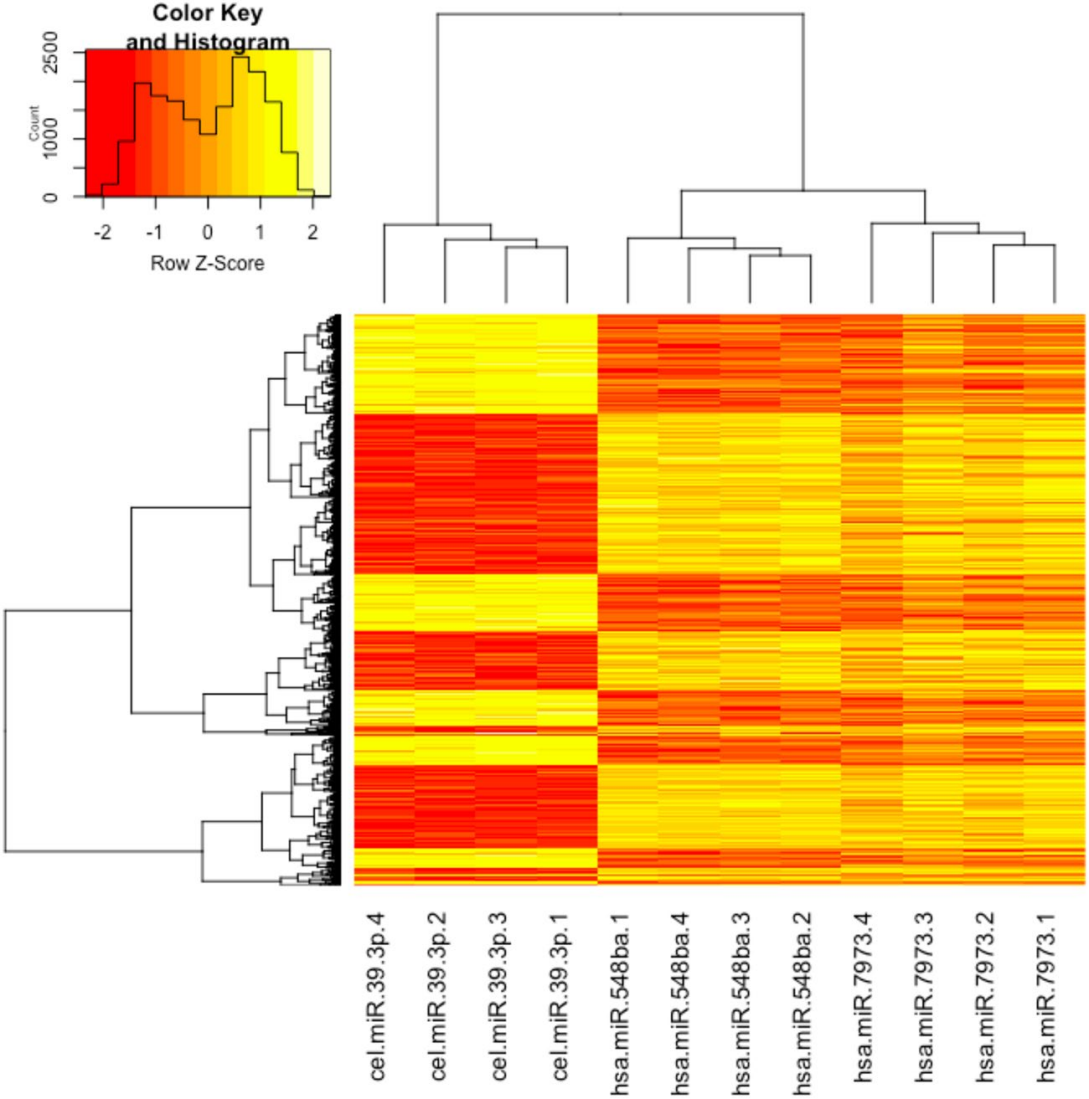
Cluster analysis of gene expression changes upon transfection of KGN cells with cel-miR-39p, hsa-miR-548ba or hsa-miR-7973 miRNA mimic. Gene expression changes were analyzed 72 h after transfection on Affymetrix microarray. Only statistically significant results are presented (adjusted p-value < 0.01, n = 4).

Cluster analysis of microarray results expectedly revealed that cells transfected with different miRNA mimics formed separate clusters (Fig. 2). However, control samples expressing cel-miR-39-3p grouped separately from samples transfected with miRNAs hsa-miR-548ba and hsa-miR-7973. This is also confirmed by the overlapping number of commonly regulated genes by the human miRNAs.

Transfection of KGN cells with hsa-miR-548ba and hsa-miR-7973 leads to the regulation of several common as well as unique signaling pathways (Supplementary Table IIIA,B). Genes differentially expressed upon hsa-miR-548ba transfection are over-represented in syndecan interactions, glycosaminoglycan and fatty acid metabolism, unfolded protein response, G protein-coupled receptor (GPCR) and ephrin signaling among others (Supplementary Table IIIA). Expression of hsa-miR-7973 in KGN cells resulted in the regulation of genes involved in signaling by netrin-1, EGFR, PDGF, and TGF-beta receptor complex, as well as pathways related to the immune system (Supplementary Table IIIB). As several genes were regulated by both miRNAs, common targeted pathways include cholesterol biosynthesis and amino acid transport across cell membrane (Supplementary Table IIIA,B).

### Bioinformatic target prediction

Microarray analysis results may partly demonstrate secondary effects on mRNA expression: gene expression changes triggered by primary miRNA targets. Therefore, to discriminate between primary and secondary target genes the differential expression results obtained from the microarray analysis method were compared to potential miRNA target mRNA-s predicted by bioinformatic algorithms. However, bioinformatic prediction may also provide false positive hits. In order to minimize such false positive findings, four target prediction programs were used, and a partial overlap of the results was used for further filtering of potential targets.

The overlap of statistically significant microarray results (adjusted p-value < 0.01) and bioinformatically predicted target genes are presented in Supplementary Table IIB and IID. Shortly, hsa-miR-548ba and hsa-miR-7973 potentially target the mRNA-s of 76 and 58 genes, respectively. Both miRNAs also share one common target mRNA of *TGFBR2* gene.

### Validation of microarray results by RT-qPCR

**Figure 3:**
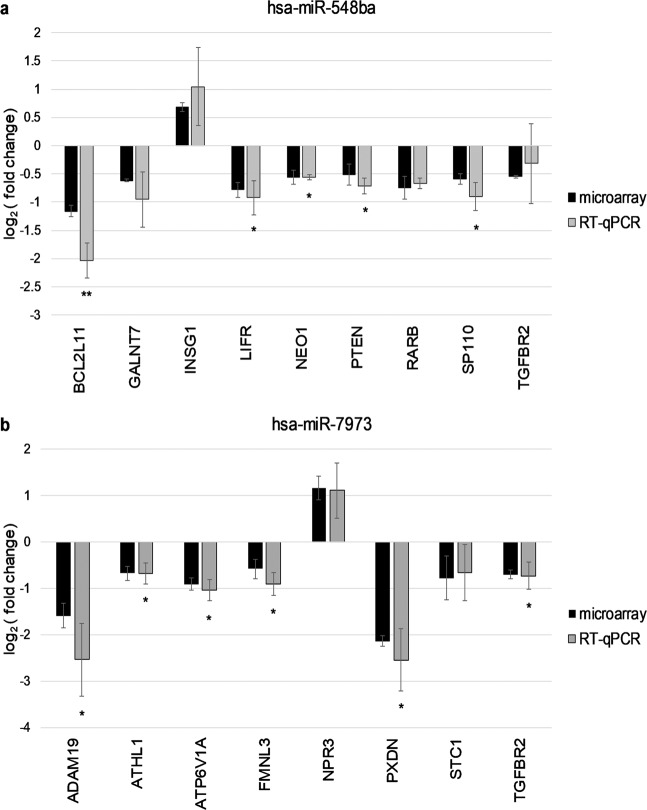
Validation of microarray results by RT-qPCR. (**a**) Potential target genes of hsa-miR-548ba and (**b**) Potential target genes of hsa-miR-7973. Gene expression change is calculated by comparing expression levels to samples transfected with control miRNA cel-miR-39-3p. Results are displayed as average fold change ±SD on log_2_ scale (*p < 0.05; **p < 0.01; Student t-test, n = 3). All presented microarray results were statistically significant (adjusted p < 0.01).

Sixteen potential target genes from the overlapping list of microarray and bioinformatic target prediction results were validated by RT-qPCR: 8 potential targets of hsa-miR-548ba, 7 of hsa-miR-7973 and their common target *TGFBR2*. The list of validated genes was selected according to published data linking the molecular functions of these proteins to their potential importance in folliculogenesis (Table 1). As a result, *BCL2L11*, *LIFR*, *NEO1, PTEN* and *SP110* were statistically significantly down-regulated at mRNA level upon cell transfection with hsa-miR-548ba mimic (p < 0.05, Fig. 3a). From the list of potential hsa-miR-7973 targets the expression levels of *ADAM19*, *ATHL1*, *ATP6V1A, FMNL3* and *PXDN* were decreased with statistical significance (p < 0.05, Fig. 3b). The expression change of the common target gene *TGFBR2* was confirmed only in case of hsa-miR-7973 transient expression (Fig. 3b).

**Table 1:** Potential target genes of hsa-miR-548ba and hsa-miR-7973 used in validation studies and their cellular functions.

Potential hsa-miR-548ba target genes			
Gene symbol	Gene name	Cellular function	Ref
*BCL2L11*	BCL2 Like 11	BCL-2 family activator of apoptosis.	^33^
*GALNT7*	Polypeptide N-Acetylgalactosaminyltransferase 7	Androgen-dependent GalNAc-transferase involved in mucin-type O-linked protein glycosylation.	^61^
*INSIG1*	Insulin Induced Gene 1	Regulates cholesterol synthesis.	^62^
*LIFR*	LIF Receptor Alpha	Growth initiation of human primordial follicles.	^24^
*NEO1*	Neogenin 1	Binds directly bone morphogenetic proteins (BMPs).	^27^
*PTEN*	Phosphatase And Tensin Homolog	Activation of primordial follicles from the dormant pool.	^25^
*RARB*	Retinoic Acid Receptor Beta	Nuclear receptor regulating the transcription of steroidogenic genes in the ovary.	^63^
*SP110*	SP110 Nuclear Body Protein	Nuclear hormone receptor coactivator, enhances retinoic acid receptor signal.	^36^
**Potential hsa-miR-7973 target genes**			
**Gene symbol**	**Gene name**	**Cellular function**	**Ref**
*ADAM19*	ADAM Metallopeptidase Domain 19	Cleaves extracellular matrix (ECM) proteins	^38^
*ATHL1*	Acid Trehalase-Like Protein 1	Cleaves ECM protein collagen.	^64^
*ATP6V1A*	ATPase H + Transporting V1 Subunit A	Involved in energy metabolism, expression correlates with human embryo quality.	^65^
*FMNL3*	Formin Like 3	Involved in cell-cell adhesion.	^42^
*NPR3*	Natriuretic Peptide Receptor 3	Maintains oocyte meiotic arrest.	^66^
*PXDN*	Peroxidasin	ECM protein with peroxidase activity.	^39^
*STC1*	Stanniocalcin 1	Regulates phosphate metabolism, potential luteinization inhibitor.	^67^
**Potential hsa-miR-548ba and hsa-miR-7973 common target gene**			
**Gene symbol**	**Gene name**	**Cellular function**	**Ref**
*TGFBR2*	Transforming Growth Factor Beta Receptor 2	Intercellular communication during ovarian follicle development.	^68^

### MicroRNA target binding validation by luciferase reporter assay

Potential miRNA targets that demonstrated significant gene expression change by microarray (p < 0.01) and RT-qPCR methods (p < 0.05) were further validated for direct miRNA:mRNA binding by luciferase reporter assay. These included hsa-miR-548ba potential target genes: *BCL2L11*, *LIFR*, *NEO1, PTEN* and *SP110*, and hsa-miR-7973 target genes: *ADAM19*, *ATHL1*, *ATP6V1A*, *FMNL3* and *PXDN*. Although *TGFBR2* gene expression change was not confirmed by RT-qPCR in hsa-miR-548ba transfected cells, binding of its 3′UTR to both miRNAs under study was nevertheless assessed.

Potential target gene *PTEN* was tested with two versions of 3′UTR lengths: longer version obtained from the UCSC Genome browser and shorter sequence used by the miRDB bioinformatical prediction program, further noted as *PTEN* long and *PTEN* short, respectively. pmirGLO-3′UTR-PTEN vector and hsa-miR-21-5p were used as a positive control pair for miRNA:mRNA binding to confirm our luciferase assay reliability, as *PTEN* has been previously shown to be a target for hsa-miR-21-5p^16^. Upon direct binding of miRNA to the 3′UTR sequence of its target mRNA, reduction in the measured luciferase signal is expected. Such suppression of luciferase signal upon hsa-miR-21-5p binding to *PTEN* 3′UTR is demonstrated in Supplementary Fig. 3 for both long and short variants.

**Figure 4:**
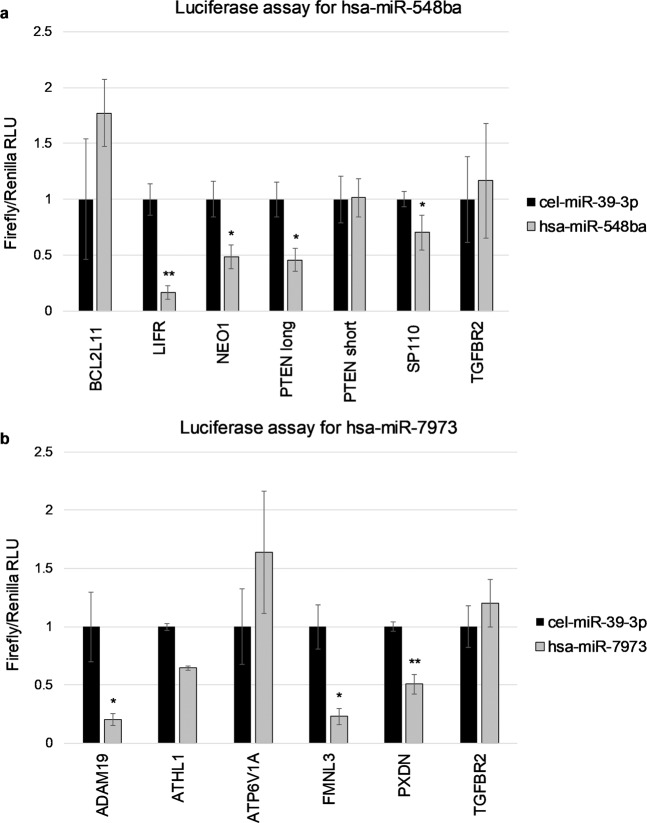
Validation of miRNA binding on the 3′UTR sequences of their potential target mRNAs by luciferase reporter assay. (**a**) hsa-miR-548ba potential target genes. (**b**) hsa-miR-7973 potential target genes. Results are shown as average normalized luciferase signal ± SEM, (*p < 0.05; **p < 0.01; Student one-tailed t-test, n = 3). RLU – relative light unit.

Luciferase assay results confirmed the direct binding of hsa-miR-548ba to *LIFR, NEO1*, and *SP110* 3′UTR sequences. Hsa-miR-548ba bound to its potential target *PTEN* 3′UTR sequence only when the longer version was used (Fig. 4a). From the tested potential targets of hsa-miR-7973 direct binding occurred on the 3′UTR of *ADAM19*, *FMNL3* and *PXDN* (Fig. 4b).

### Effect of hsa-miR-548ba and hsa-miR-7973 transient expression on the endogenous protein levels of their target genes

The endogenous expression levels of target proteins were assessed at two time-points: 72 h (coinciding with the highest effect of miRNA transfection on target gene mRNA levels) and 96 h after transfection with miRNA mimics. As we are unable to account for effects triggered by long-term culture, secondary miRNA targets and the kinetics of each endogenous protein expression and stability, the difference in protein levels between the time-points and between cells transfected with miRNAs under study vs control miRNA cel-miR-39-3p were investigated. We observed different trends in endogenous protein expression levels for PTEN between cells transfected with has-miR-548ba and cel-miR-39-3p: while in former samples the expression level increased 4.34 times, in control cells the levels decreased 1.28 times from 72 h to 96 h (Supplementary Fig. 4A).

The level of PXDN, a potential target of hsa-miR-7973, decreased from 72 h to 96 h by 2-fold. In comparison, the level of PXDN was up-regulated 2 times during the observed time-frame in control samples. A trend for at least 2-fold change in protein expression between time-points and opposite direction of expression levels between cells transfected with hsa-miR-7973 and cel-miR-39-3p was also observed for TGFBR2. The expression of ATHL1 did not change in control experiment, while it increased 2-fold from 72 h to 96 h in cells transfected with hsa-miR-7973 (Supplementary Fig. 4B).

Protein expression remained below detection limit for BCL2L11, LIFR and ADAM19.

### Influence of FSH stimulation on the expression of miRNAs and their host genes in primary granulosa cells

Hsa-miR-548ba and hsa-miR-7973 genes are located in the intronic regions of *FSHR* and *CYP19A1* genes, respectively. To test the correlation between miRNA and its host gene expression levels, long-term culture of primary human CGC and MGC was used. Cells were cultured in parallel with or without FSH stimulation (1 IU/ml) that is well known to increase the expression levels of both, FSHR and CYP19A1^13^.

**Figure 5:**
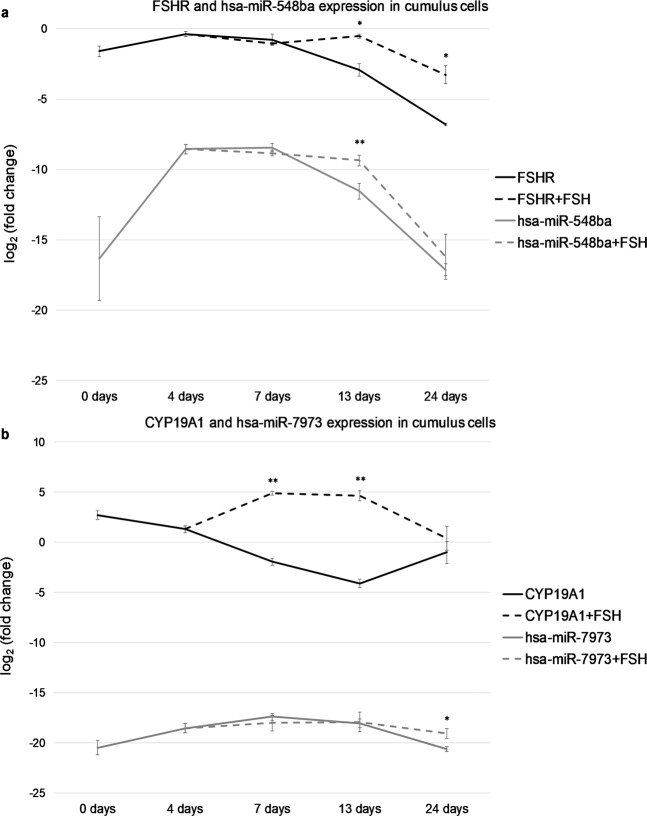
miRNA and host gene expression levels in primary human cumulus granulosa cells. mRNA and miRNA expression levels were normalized to SDHA or hsa-mir-132-3p, respectively. (**a**) hsa-miR-548ba and FSHR expression and (**b**) hsa-miR-7973 and CYP19A1 expression. Results are displayed as average fold change ± SD on log_2_ scale (*p < 0.05; **p < 0.01; Student t-test between cells exposed to 1 IU/ml FSH vs non-treated cells, n = 3).

Hsa-miR-548ba was expressed well in correlation with its host gene *FSHR* both under stimulated and non-stimulated conditions similarly in both cell populations (Fig. 5a and Supplementary Fig. 5a). While the expression level of *CYP19A1* mRNA increased upon treatment of cells with FSH, such increase was not observed for the expression of hsa-miR-7973 in neither of the cell populations (Fig. 5b and Supplementary Fig. 5b). However, the expression of hsa-miR-7973 remained at a steady level up to 24 days in the presence of FSH in the culture medium in comparison to cells without FSH treatment. The latter result was statistically significant only in CGC (Fig. 5b).

## Discussion

We present the first identification and validation results of hsa-miR-548ba and hsa-miR-7973 target genes.

The miRNAs of interest: hsa-miR-548ba and hsa-miR-7973, were both identified for the first time from human ovaries by deep sequencing approach of primary granulosa cells^11^. In the experiments of the current study we first used granulosa tumor-like KGN cell line as a granulosa cell model^15^. KGN cells express the functional FSH receptor and high levels of aromatase, encoded from miRNA hsa-miR-548ba and hsa-miR-7973 host genes *FSHR* and *CYP19A1*, respectively. KGN cell line has also been previously used for miRNA target identification and luciferase assay studies for direct miRNA:mRNA interactions^17^.

MicroRNAs are estimated to regulate 60% of all protein-coding genes and a single miRNA may regulate up to 400 different target mRNAs^18^. Our microarray results demonstrated approximately 1,500 differentially expressed genes upon transfection with either of the miRNAs under study, while 459 genes were regulated specifically by hsa-miR-548ba and 537 by hsa-miR-7973. Gene ontology analysis from hsa-miR-548ba regulated genes showed enrichment in fatty acid metabolism and ephrin signaling pathways and over-expression of hsa-miR-7973 showed enrichment in TGF beta receptor complex and immune system pathways. Commonly regulated genes by these two miRNAs targeted the cholesterol biosynthesis pathway. Cholesterol is a major component for steroidogenesis and sources of cholesterol for ovarian granulosa cells include plasma lipoproteins and de novo-synthesis^19^. Therefore, the regulation of cholesterol biosynthesis pathway by the miRNAs under study may influence steroid hormone production, contributing to the follicular developmental stage-dependent estradiol and progesterone production.

Although, miRNAs may regulate several hundreds of target genes, microarray results do not differentiate primary targets from secondary regulatory effects: i.e. the genes regulated by primary miRNA targets. Such secondary regulatory effects are common in over-expression conditions and large data-sets obtained by genome-wide methods^20^. The large number of commonly regulated genes by both miRNAs analyzed in the present study can be also explained by secondary effects on gene expression, as the over-expression of miRNAs upon transfection may lead to the saturation of RNA silencing complex (RISC) which results in altered gene expression regulation^21^. To distinguish primary miRNA targets from secondary ones, microarray results were compared to bioinformatically predicted target genes.

For direct miRNA:mRNA binding studies luciferase assay reporter vectors were cloned with potential target gene 3′UTR sequences. In this study full length 3′UTR sequences were used, except for *PTEN* in case of which bioinformatic prediction algorithms do not use consistent 3′UTR sequence lengths and thus two different sequences were analysed. We chose to test full-length sequences for most of the target genes as we lacked the knowledge of functional miRNA target regions and the exact biding site identification within each potential target gene was not the goal of the current study. Additionally, testing a fragment of 3′UTR may easily lead to false results due to the different secondary structure from the full length sequence^22^. Direct binding assay confirmed *LIFR*, *PTEN*, *NEO1* and *SP110* as hsa-miR-548ba targets. For target gene *PTEN* direct binding occurred only with the longer 3′UTR sequence, although bioinformatic algorithms used in this work predicted target sequences into the shorter 3′UTR isoform. Such results can be explained by the differences in the secondary structure mentioned above. Moreover, bioinformatical programs might not predict all of the potential non-canonical miRNA target sequences.

The change in endogenous target protein expression levels was observed for PTEN (in case of transfection with hsa-miR-548ba), as well as for PXDN, TGFBR2 and ATHL1 (targets of hsa-miR-7973) in comparison to cells transfected with cel-miR-39-3p. As the regulation kinetics is expectedly different for each protein, the molecular mechanism behind the up-regulation of PTEN and downregulation of other targets cannot be explained based solely on our experiments. We demonstrate that the expression of the miRNAs under study influence the protein levels of their target genes, however, the changes in protein expression cannot be uncoupled from the effects of secondary targets and signaling pathways activated upon transient miRNA transfection.

The confirmed targets for hsa-miR-548ba LIFR, PTEN and NEO1 all play a role in the early stages of follicle development. Leukemia inhibitory factor (LIF), the ligand for LIFR, promotes primordial to primary follicle transition in the rat ovary^23^. In humans, the role of LIF and its receptor in the ovary is not well characterized, but LIF and LIFR mRNA expression in human ovarian samples are consistent with the concept that LIF might be involved in growth initiation of human primordial follicles through its receptor^24^. The recruitment of primordial follicles is regulated by highly controlled mechanisms and the critical role is played by PI3K-Akt signaling pathway. This pathway is negatively regulated by PTEN and it has been shown that in mice lacking *PTEN* the entire primordial follicle pool becomes activated^25^. The levels of PI3K-Akt signaling pathway components in granulosa cells have a correlation with oocyte competence in bovine as decreased PTEN expression and increased expression of PTEN downregulating miRNAs correlate with the number of high-quality oocytes^26^.

NEO1 is a receptor for repulsive guidance molecules, netrins, and is involved in bone morphogenetic protein (BMP) signaling pathways by directly binding BMPs^27^. Moreover, NEO1 activates RAC1-PI3K-AKT signaling pathway in human gastric cancer cells^28^. In mouse ovary, Rac1 is involved in primordial follicle formation by inducing nuclear transport of SMAD3^29^. As mentioned above, PI3K-AKT signaling pathway have crucial roles to balance follicle growth suppression, activation and progression also in humans^30^ and BMPs have important role in follicle development including primordial germ cell development, oocyte-somatic cell interactions and modulating cumulus-oocyte complex (COC) formation and expansion^31^.

Moreover, direct hsa-miR-548ba targets *PTEN*, *NEO1* and potential secondary target *BCL2L11* are also involved in regulating apoptosis^32–34^. In cattle, follicle atresia is regulated by LIF-STAT3 pathway which can be reversed with FSH administration^35^. In the current study, the transient expression of hsa-miR-548ba did not lead to changes in overall cellular viability during 72 h. Therefore, the role of hsa-miR-548ba in modulating apoptotic pathways in the human ovary needs to be investigated further.

The role of SP110 in the ovary is less clear. It has been suggested that SP110 may function as a nuclear hormone receptor transcriptional coactivator via binding to the retinoic acid receptor alpha (RARA) response element^36^. Retinoids are essential for steroid production and it has been demonstrated that bovine CGCs express active RARA^37^.

The confirmed targets of hsa-miR-7973 according to our study were *ADAM19*, *PXDN* and *FMNL3*, all of which have been shown to be involved in extracellular matrix (ECM) modulation and cell-cell interactions^38,39^. ADAM19 is involved in ECM remodeling by cleaving ECM proteins, growth factors and cytokines like neuregulin^38^. Neuregulin 1 is expressed by granulosa cells and regulates the meiotic resumption time of the oocyte leading to proper oocyte developmental competence in cultured COCs^40^.

Other two confirmed targets of hsa-miR-7973, PXDN and FMNL3, have not been studied in relation to folliculogenesis, but are involved in inter-cellular and cell-ECM contacts. PXDN is a peroxidase previously investigated in the context of ovarian cancer where the knockdown of this gene inhibits cellular proliferation, invasion and migration^39^. FMNL3 protein is involved in cytoskeletal organization, cell morphology, migration and cell-cell adhesion^41,42^.

Genes demonstrating negative results in luciferase reporter assay can be considered as secondary targets of the miRNAs of interest, but one should consider that the experiments were performed with reporter vectors and in cell line conditions that may not fully reflect physiological conditions in primary cells. MiRNA target recognition may require multiple transacting factors available only in specific cellular contexts. Also the alternative polyadenylation of 3′UTRs in reporter vectors may increase the number of false-negative results^43^. In addition, many possible target genes were excluded in this study due to statistically non-significant results from microarray gene expression analysis and were thus not tested in the direct binding assay. Therefore, due to the stringent filtering approach used in the current study, the list of validated target genes for these two miRNAs of interest is not final.

Another goal of the study was to investigate, whether the expression of miRNAs coincides with the levels of their host genes. This may give indication on the biogenesis of the miRNAs under study, as intronic miRNAs may be generated alongside with the pre-mRNA intron splicing mechanism^44,45^. As KGN cells do not endogenously express detectable levels of hsa-miR-548ba and hsa-miR-7973, primary cell cultures of human stimulated cumulus and mural granulosa cells were used. We demonstrate that the miRNAs under study are stably expressed in the long-term culture of human primary granulosa cells for 24 days. It is clear from our results that the regulation of expression of the two miRNAs is achieved by different mechanisms. Hsa-miR-548ba is expressed in correlation with the mRNA of its host gene *FSHR*, both in presence or without FSH in culture medium. Such co-expression indicates the possibility that the miRNA is excised from the intron of *FSHR* pre-mRNA. However, there is no substantial co-expression of hsa-miR-7973 and its host gene *CYP19A1*. Hsa-miR-7973 is stably expressed over time, indicating a different mechanism of regulation: possibly an alternative promoter for the miRNA gene. However, additional experiments are necessary to draw firm conclusions about the biogenesis of these miRNAs.

Overall, we identified and confirmed several targets for hsa-miR-548ba and hsa-miR-7973 and showed that the endogenous expression of these miRNAs is maintained in long-term culture of primary granulosa cells. According to the previously known functions of their target genes we propose undescribed regulatory roles for these miRNAs in granulosa cell gene expression and in follicle development.

## Materials and Methods

### Cell culture of KGN cell line

Granulosa tumor-like cell line KGN was used as a model for cell line experiments^15^. Cells were grown in L-glutamine-containing Dulbecco’s Modified Eagle’s medium with 10% fetal bovine serum, 100U/ml penicillin and 0.1 mg/ml streptomycin on 60 × 15 mm culture plates at 37 °C and 5% CO_2_. Cells were harvested with 0.05% trypsin and 0.02% EDTA (all solutions from Naxo, Tartu, Estonia).

### Isolation of primary granulosa cells

The study was approved by the Ethics Committee of the University of Tartu and conducted according to the guidelines of the Declaration of Helsinki. An informed consent was obtained from all participants. For miRNA expression validation granulosa cells were collected from 8 women undergoing ovarian stimulation according to the GnRH antagonist protocol (described below), ovarian puncture (OPU) and oocyte intra-cytoplasmic sperm injection due to male-factor infertility. The general characteristics of the study participants were as follows (mean ± SD): age 31.0 ± 6.2 years, BMI 22.9 ± 3.5, total FSH amount used 1528.0 ± 653.8 IU, the number of retrieved oocytes was 12.0 ± 9.1, of which 75.0 ± 22.2% were considered mature according to the visibility of the first polar body. For the long-term granulosa cell culture experiments samples were collected anonymously from consecutive *in vitro* fertilization patients and pooled for the reduction of inter-patient variability. Each pool consisted of granulosa cells from ≥ 10 patients.

Ovarian hormonal stimulation was conducted according to the GnRH antagonist (Cetrotide, Merck Serono, Geneva, Switzerland) protocol with the administration of recombinant FSH (Gonal-F, Merck Serono, or Puregon, Merck Sharp & Dohme Corp., Whitehouse Station, NJ, USA). All patients underwent OPU of follicles ≥ 15 mm in size after 36 h of hCG administration (Ovitrelle, Merck Serono).

MGCs were obtained from follicular fluid after OPU following the manual removal of cumulus-oocyte complexes and CGC aggregates devoid of the oocyte. The fluid from all follicles of a patient was pooled, centrifuged at 450 *g* for 10 minutes, followed by supernatant removal. The cells were separated on a 50% density gradient of PureSperm 100 (Nidacon; Mölndal, Sweden) in Universal IVF Medium (Origio; Jyllinge, Denmark), washed three times in Universal IVF Medium at 37 °C, depleted of CD45-positive leukocytes according to the manufacturer’s suggested protocol (DynaMag and Dynabeads; Life Technologies, Carlsbad, CA, USA), lysed with QIAGEN miRNeasy Mini kit lysis buffer (QIAGEN, Hilden, Germany), and stored in liquid nitrogen.

CGC were collected 4 h after OPU during oocyte denudation lasting up to 5 min with type IV-S hyaluronidase extracted from bovine testes (Sigma-Aldrich, St-Louis, MO, USA) and diluted in Sperm Preparation Medium (Origio). The CGC from all oocytes irrespective of their maturity were pooled per patient and centrifuged at 450 g for 5 min and the supernatant was discarded. For direct analysis of miRNA content, the cells were lysed and stored as described above.

### Cumulus granulosa cell culture

For primary cell culture experiments the collected cumulus cells were pooled (pools of 10–30 patients). Cells were counted and 500 000 cells were plated onto Matrigel-covered 6-well plates (Corning, New York, NY, USA) in DMEM/F12 growth medium (Lonza AG, Basel, Switzerland) with 20% KnockOut Serum Replacement (KSR) (Thermo Scientific, Waltham, MA, USA), 1% penicillin/streptomycin (Naxo, Tartu, Estonia) and Primocin (100 μg/ml) (InvivoGen, San Diego, CA, USA). IGF2 (50 ng/ml) (Bio-Techne, Minneapolis, MN, USA) and FGF2 (8 ng/ml) (Enantis, Brno-Bohunice, Czech Republic) were added to the growth medium. The effect of FSH (1 U/ml) (Gonal-F, Merck Serono) was tested starting from day 4, because it has been shown that GCs are not responsive to hormones right after collection^46^. Growth medium was changed daily. Cells were passaged using TrypLE (Thermo Scientific), half of the cells were re-plated and half were lysed using QIAzol lysis reagent (QIAGEN). All cell culture experiments were repeated three times.

### Cell line transfection

For microRNA transfection Lipofectamine RNAiMAX reagent (Invitrogen, Carlsbad, CA, USA) and miRCURY LNA miRNA mimics hsa-miR-548ba, hsa-miR-7973 and control miRNA with no mammalian homologue cel-miR-39-3p^47,48^ were used according to manufacturer’s protocol (Exiqon, Vedbaek, Denmark). MicroRNA mimics were transfected at previously optimized concentration of 12.5 nM and 72 h time-point after transfection was tested.

For miRNA and luciferase vector co-transfection Lipofectamine 2000 reagent (Invitrogen) and previously specified miRCURY LNA miRNA mimics were used according to manufacturer’s protocol. MicroRNA final concentration in culture medium was 12.5 nM or 50 nM, while 100 ng of vector was transfected.

### Cytotoxicity analysis

MicroRNA cytotoxicity analysis was performed using CytoTox-Glo Cytotoxicity Assay (Promega Corporation, Madison, WI, USA) according to the user manual. Five thousand cells were plated in each well of a white Greiner CELLSTAR 96-well plate (Sigma-Aldrich) and transfection was performed 24 h after plating. Non-transfected control samples were measured at 0, 24, 48, and 72 h. The cytotoxicity of cells transfected with either hsa-miR-548ba, hsa-miR-7973 or cel-miR-39-3p mimics was measured at 24, 48 and 72 h post-transfection. The above-mentioned assay uses a luminogenic peptide substrate to measure dead-cell protease activity, which is released from cells upon losing their membrane integrity. Luminescence was measured in two steps with Tecan GeniosPro luminometer (Tecan, Männedorf, Switzerland). Firstly, luminescence from dead cells and secondly luminescence from all cells after lysis was detected. Results were calculated as luminescence from all lysed cells minus luminescence from dead cells. All samples were measured in triplicates and the average value of three independent experiments was used in data analysis.

### RNA extraction

Cells were lysed in QIAzol lysis reagent (QIAGEN). For total RNA extraction miRNeasy Mini Kit (QIAGEN) was used. In addition, small fraction RNA ( ≤ 200 nucleotides) extraction was performed separately using RNeasy Mini Elute Cleanup Kit (QIAGEN). Both total and small RNA extraction was performed according to the user manual. RNA concentrations were measured using NanoDrop 2000c spectrophotometer (Thermo Fisher Scientific, Dreieich, Germany).

The quality of all RNA samples analyzed on Affymetrix microarray was evaluated using the Agilent 2100 Bioanalyzer system (Agilent Technologies, Waldbronn, Germany). All RNA samples were of high quality, with the RNA Integrity Number values between 9.2–9.8.

### Affymetrix microarray

Affymetrix microarray was performed at the Array and Analysis Facility at Uppsala Biomedical Center, Sweden. From each sample 250 ng of total RNA were used to generate amplified and biotinylated sense-strand cDNA according to the GeneChip WT PLUS Reagent Kit User manual (Affymetrix, Santa Clara, CA). GeneChip Human Gene 2.0 ST Arrays were hybridized for 16 h in 45 °C incubator, rotated at 60 rpm. Arrays were washed and stained using the Fluidics Station 450 and scanned using GeneChip Scanner 3000 7 G. Affymetrix microarray data is available at Gene Expression Omnibus repository, accession number GSE122731.

### Reverse transcription

For cDNA synthesis from mRNA SuperScript III First Strand Synthesis SuperMix (Invitrogen) was used according to user manual. 1 μg of cell line and 500 ng of primary granulosa cell RNA was used as input.

For microRNA detection cDNA was synthesized using miRCURY LNA^TM^ Universal RT microRNA PCR Universal cDNA Synthesis Kit III (Exiqon). 200 ng of small fraction RNA was used as input.

### Primer design

Primers used for RT-qPCR analysis were designed using NCBI primer-BLAST (http://www.ncbi.nlm.nih.gov/tools/primer-blast/). Preferred primers spanned exon-exon junctions and amplified all splice isoforms of the given gene. Primer sequences are presented in Supplementary Table IA.

Primers for amplifying the 3′UTR sequences of potential miRNA target genes were designed in Benchling (https://benchling.com/). Primer binding specificity was tested using NCBI primer-BLAST (Supplementary Table IB).

For the amplification of miRNAs, commercially available primers were used (Exiqon).

### RT-qPCR

The RT-qPCR analysis was carried out using SDS 2.3 software in 7900HT Fast Real-Time PCR System (Applied Biosystems, Foster City, California). For the detection of mRNA expression, Platinum SYBR Green qPCR SuperMix-UDG (Invitrogen) or Platinum SYBR Green qPCR SuperMix-UDG with ROX (Invitrogen) was used. cDNA from small RNA fraction was amplified by EXILENT SYBR Green Mastermix (Exiqon) according to the user manual. Each sample was run in triplicates on a 384-well plate up to 40 amplification cycles. The specificity of amplified PCR products was determined by melt curve analysis.

### Luciferase reporter vector cloning and luciferase assay

The full length 3′UTR of hsa-miR-548ba potential target genes *BCL2L11*, *LIFR*, *NEO1*, *PTEN*, *RARB*, *SP110*, hsa-miR-7973 target genes *ADAM19*, *ATHL1*, *ATP6V1A*, *PXDN*, *FMNL3*, and the common target for both miRNAs *TGFBR2* were cloned into pmirGLO Dual-Luciferase vector (Promega) downstream of the Firefly luciferase gene. Target gene 3′UTR sequences were obtained from UCSC Genome Browser (https://genome.ucsc.edu/) with an exception of *FMNL3* where shorter isoform of 3′UTR was used. Bioinformatic prediction program TargetScan 7.1^49^ uses *FMNL3* 3′UTR isoform with length 7,874 nt, while in UCSC Genome Browser the length is 9,316 nt. Moreover, for *PTEN* also a shorter version of 3′UTR isoform was cloned which is used by miRDB bioinformatical target prediction program^50^. Potential target gene 3′UTR sequences and predicted miRNA binding sites are available in Supplementary Material. Primers for amplifying full length 3′UTR from genomic KGN cell line DNA and restriction enzymes used for cloning are presented in Supplementary Table IB.

Validation of hsa-miR-548ba and hsa-miR-7973 direct binding to target gene 3′UTR was performed by using Dual-Glo Luciferase assay system (Promega) according to the user manual. Luciferase signal was measured 24 h after miRNA and vector co-transfection. The relative luciferase activities were determined by calculating the ratio of Firefly luciferase activities over Renilla luciferase activities. All experiments were repeated three times as triplicates on a white Greiner CELLSTAR 96-well plate (Sigma-Aldrich). Cel-miR-39-3p was used as a negative control.

### Design of peptides for targeted proteomics

Synthetic peptides for targeted proteomics were designed based on the total proteome analysis of KGN cell lysate (described in Supplementary Materials). Two peptides per protein were selected. In addition, GAPDH was selected as an endogenous control. Peptides with the highest intensity in the library and without methionines and cysteines were preferred for the targeted analysis. The sequences of the synthetic peptides are presented in Supplementary Table IC.

### Sample preparation for targeted proteomics analysis

KGN cells were transfected with hsa-miR-548ba, hsa-miR-7973 or cel-miR-39-3p mimics in triplicates as described above. After 72 h and 96 h post-transfection the cells were removed from the plate by trypsin, washed with PBS, and centrifuged at 1,700 g.

The pelleted cells were suspended in 10 volumes of 4% SDS, 100 mM Tris-HCl pH 7.5, 100 mM DTT lysis buffer. Samples were heated at 95 °C for 5 min, followed by probe sonication (Bandelin, Berlin, Germany) (60 × 1 s pulses, 50% intensity). Unlysed material were cleared with centrifugation at 14,000 g for 10 min, although, lysis was noted to be nearly complete. 30 µg of protein was precipitated with 2:1:3 (v/v/v) methanol:chloroform:water. Protein pellets were suspended in 25 µl of 7 M urea/ 2 M thiourea 100 mM ammonium bicarbonate (ABC) solution, followed by disulfide reduction and cysteine alkylation with 5 mM DTT and 10 mM chloroacetamide for 30 min each at room temperature. Proteins were predigested with 1:50 (enzyme to protein) Lys-C (Wako Chemicals, Neuss, Germany) for 4 h, diluted 5 times with 100 mM ABC and further digested with trypsin (Sigma Aldrich) overnight at room temperature. Peptides were desalted with in-house made C18 StageTips.

### Targeted LC/MS/MS analysis

Peptides were injected to an Ultimate 3000 RSLCnano system (Dionex, Sunnyvale, CA, USA) using a 0.3 × 5 mm trap-column (5 µm C18 particles, Dionex) and an in-house packed (3 µm C18, Dr Maisch, Ammerbuch-Entringen, Germany) analytical 50 cm × 75 µm emitter-column (New Objective, Woburn, MA, USA). Peptides were eluted at 250 nl/min with an A to B 10–45% 60 min gradient (buffer A: 0.1% formic acid, buffer B: 80% acetonitrile + 0.1% formic acid) to a Q Exactive Plus (Thermo Fisher Scientific) MS/MS using a nano-electrospray source (positive mode, spray voltage of 2.6 kV). The MS was operated in a scheduled parallel reaction monitoring (PRM) mode by isolating and fragmenting only peptides from the selected proteins within ± 4 minute windows of their retention times. Retention time scheduling was calibrated using the indexed retention time (iRT) method^51^. MS/MS isolation window was 1.0 m/z and scans were performed at a resolution setting of 17,500. Ion target value of 2e5 and fill time of 60 ms were used. Normalized collision energy was set at 26.

### Data analysis and statistics

#### Affymetrix microarray data analysis

Affymetrix microarray raw data was normalized in Expression Console, provided by Affymetrix (http://www.affymetrix.com), using the robust multi-array average (RMA) method. Gene annotations were drawn from the probeset ID-s of the HuGene-2_0-st chip. Differential gene expression analysis was carried out in the statistical computing language R (http://www.r-project.org) using packages available from the Bioconductor project (www.bioconductor.org)^52^. To detect differentially expressed genes between samples transfected with hsa-miR-548ba or hsa-miR-7973 and cel-miR-39-3p groups, an empirical Bayes moderated t-test was applied using the “limma” package^53^. The p-values were adjusted for multiple testing according to the method of Benjamini and Hochberg^54^. Statistical significance level was set at adjusted p < 0.01.

For cluster analysis of microarray data genes were ordered by the highest fold change expression difference upon hsa-miR-548ba transfection and Bioconductor package “heatmap” was used.

#### Gene ontology analysis

Analysis of over- and under-representation of Reactome pathways (version 58) was performed in the Panther classification system^55^. Genes differentially expressed (adjusted p < 0.01) on Affymetrix microarray upon transfecting KGN cells with either hsa-miR-548ba or hsa-miR-7973 mimics compared to the transfection with cel-miR-39-3p were used as input. Fisher’s exact test with Benjamini and Hochberg false-discovery rate (FDR) correction was used to test the statistical significance of the results and over- or under-represented pathways obtaining FDR < 0.05 are reported.

#### MicroRNA target gene prediction

For miRNA bioinformatic target prediction four web based programs were used (DIANA microT v 3.0^56^, microT CDS v 5.0^57^, TargetScan 7.1^49^ and miRDB^50^). Gene was considered as a potential miRNA target in case it was predicted by at least two programs out of four and its gene expression fold change according to microarray analysis was ≥ log_2_(|0.3|).

#### RT-qPCR

The gene-specific mRNA expression Ct values were normalized against *GAPDH* expression and miRNA expression levels were normalized for internal control hsa-miR-132-3p using the method of relative quantification by Pfaffl^58^. Several control small RNAs were tested for the stability of their expression levels and hsa-miR-132-3p was chosen due to the least variability in its expression between patients and granulosa cell populations (Supplementary Methods). In primary CGC culture experiments *SDHA* was used as an endogenous control for the normalization of mRNA levels. Experiments were run in technical triplicates three times. Results are shown as average ± SD (standard deviation). Statistical significance was calculated by two-tailed Student t-test in Microsoft Office Excel 2017. Statistical significance level was set at p < 0.05.

#### Luciferase assay

The relative luciferase activities were determined by calculating the ratio of Firefly luciferase relative light units (RLU) over Renilla luciferase RLU. All experiments were run independently three times as triplicates. Results are shown as average ± SE (standard error). Statistical significance was calculated by one-tailed Student t-test in Microsoft Office Excel 2017. Statistical significance level was set at p < 0.05.

#### LC/MS/MS data analysis

MS raw files were analysed with the Skyline software^59^. Spectral library was created from Mascot (Matrix Science, Woburn, MA, USA) database search results derived from measuring synthetic peptides (JPT Technologies, Berlin, Germany) with data-dependent LC/MS/MS mode. Only y-ions (starting from ion 4, y4 up to last ion −1) with charge states + 1 and + 2 were allowed. All extracted ion chromatogram (XIC) integrations were manually inspected for correct peak picking. Fragment XIC traces with strong interference and erroneously picked peaks (mass errors >  ± 20 ppm, lack of fragment coelution) were removed.

For the comparison of protein expression levels between samples, peak areas referring to the same proteins were summed. Results were transferred to MaxQuant Perseus module^60^, log_2_-transformed and filtered for at least 50% valid values in each group. Missing values were imputed by down-shifting and compressing the measured intensity distributions by 1.8 and to 0.3 standard deviation units, respectively, thereby simulating intensity measurements on the measurement threshold. All values were then normalized to GAPDH expression. Two comparisons per protein were made: (a) change in expression level between 96 h and 72 h after miRNA transfection, and (b) change in expression between cells transfected with either hsa-miR-548ba or hsa-miR-7973 and cel-miR-39-3p. Difference in the level of protein expression is reported, if the expression change between time-points is > 2-fold and if opposite direction of expression change is observed between the cells transfected with the miRNAs under study and the control miRNA.

## Supplementary information

Supplementary Information.

Supplementary Information2.

Supplementary Information3.

Supplementary Information4.
