# Supplementary material for: Target prediction and validation of microRNAs expressed from FSHR and aromatase genes in human ovarian granulosa cells
Source: Sci Rep. 2020 Feb 10;10:2300. doi: 10.1038/s41598-020-59186-x (PMC7010774; doi:10.1038/s41598-020-59186-x)
Supplement: Supplementary file 1 — Supplementary Information. [file 41598_2020_59186_MOESM1_ESM.docx]

**Target prediction and validation of microRNAs expressed from FSHR and aromatase genes in human granulosa cells**

Ilmatar Rooda^1,2^, Kati Hensen^3^, Birgitta Kaselt^1^, Sergo Kasvandik^4^, Martin Pook^3^, Ants Kurg^3^, Andres Salumets^2,5,6,7^, Agne Velthut-Meikas^1,2,*^

^1^Department of Chemistry and Biotechnology, Tallinn University of Technology, Tallinn, Estonia

^2^Competence Centre on Health Technologies, Tartu, Estonia

^3^Institute of Molecular and Cell Biology, University of Tartu, Tartu, Estonia

^4^Proteomics Core Facility, Institute of Technology, University of Tartu, Tartu, Estonia

^5^Institute of Clinical Medicine, Department of Obstetrics and Gynecology, University of Tartu, Tartu, Estonia

^6^Institute of Biomedicine and Translational Medicine, Department of Biomedicine, University of Tartu, Tartu, Estonia

^7^Department of Obstetrics and Gynecology, University of Helsinki and Helsinki University Hospital, Helsinki, Finland

**Supplementary Material**

Target gene 3’UTR sequences and bioinformatically predicted target sites for miRNAs hsa-miR-548ba or hsa-miR-7973. Four target prediction programs were used: DIANA microT v 3.0, microT CDS v5.0, TargetScan 7.1 and miRDB. Red underlined sequences represent seed sequences predicted by both TargetScan and miRDB, green underlined sequences represent microT CDS v.5.0 predicted target sequences, blue underlined sequences represent only TargetScan predicted seed sequence location and yellow marks seed sequences predicted only by miRDB. Hsa-miR-548ba potential target gene 3’UTRs are: a) *BCL2L11*, b) *LIFR*, c) *NEO1*, d) *PTEN*, e) *RARB* and f) *SP110*. Hsa-miR-7973 potential target gene 3’UTRs are: g) *ADAM19*, h) *ATHL1* i) *ATP6V1A*, j) *FMNL3* and k) *PXDN*. Potential target gene for both hsa-miR-548ba and hsa-miR-7973 3’UTR is: k) *TGFBR2*.

| 1. *BCL2L11* 3’UTR sequence with underlined bioinformatically predicted hsa-miR-548ba target sites. microT CDS v5.0 predicted target sites are marked in green: positions 144-163, 656-675, 2310-2328 and 2696-2717. TargetScan and miRDB commonly predicted seed sequences are marked in red: positions 2322-2328 and 2712-2718. | |
| --- | --- |
| 1 CAGGTTCTTT GCGGAGCCGA GATACCATGC AGACATTTTG CTTGTTCAAA CCAACAAGAC  61 CCAGCACCGC GGTCTCCTGG TGCCATTATT ATGCAGCCAG CGGTTCTCTT GTGGAGGGGG  121 CAGGTGACGT TTCAGAAGAC ACCG**AGCTGG ATGGGACTAC CTT**TCTGTTC ATCACCACAC  181 AGCAGAATTT CTAATGGAAG TTTGTTGTGA ATGTAAAGGA GGGAGCATTC TTTGCTTTTT  241 AATATACAAA CCATGGTTTT TTGGAGCAGG ATTTTGTGTA AGAATGGTGT TTACATGCAG  301 TGTGTTTTCC CCCTCACCTT CAATAAGGTT TTTCAAAAAG GAAATGGAAA CTTTTTAACC  361 AATTTGTGAA TAACTTTTGT ATTAAAATTT TAAGAACCTA CGGCCTATTC TCAGAGGATT  421 ATGTAACCCC TGCAGTGGAA ACTGAGCCAG CTAACTTAAA AAGCTGCCTT AGTTTATTTT  481 TAGAGATTAC AGAATTTTTA AACAGGGAGA CGTGTGATAT ACTCCCTCCC TTCCCTACTA  541 TTGCCTCTCT GACCTTTTTA AATTATTTTT AATACCAAAA GAGTTCTTTT GAAATGGAAC  601 TGATTAAAAG GGCAGAGGGT CTGTTGCCAG CCTGCATTGA TATACCAGTC CCATTT**GTAA**  661 **ATATTTACGT ACCTT**TATAA ATTCAGTTGC ATCTGTGGCA AAATTTCAGA CTATTTTTGC  721 GTCTTTCCTC ATCACTTTTT GTGATGCAAC TCCAGTCTGG ACTCAGATGC ATAGATTTGG  781 TCCAGTGTAT TTTCATGATA AAGTGAAATT GAGTCAGAAC AAGAGTTAAT ATCTGCCTGT  841 ATCTTGCACA GTTCGAGCGA TCTGTTATTA ACTGGGAAGC ATTTGGTGTT GGTTTTCATT  901 CCATTTCGAC GAGCATGTTA TTGGGAAGTA TTCTGAAGAG GCAATAGCAG TAATAACAAC  961 AGACTTAAGT GCTACGCCCC TTTGTGCTGC TGGCTTTTCT GGTTGCAGGC TTTCCCATGG  1021 TCACAGGATG CACTGTCAGC ATCAGGTCCC AGAGGGCCAC CGTGTCCATT ACAGCAGAGT  1081 CCAGCTGCAG CATCCAGCTC ACGCCCTCAT GGGAATTGGC ACAGGCCTGG GGCAGGGCTT  1141 CTGATGGCCA TTTGCTTGGC CTCCTGCATT TTAGTCCAAC TCACAGTCCA CTAGCTTCAC  1201 TCCTTTAAAT TCACTTTGAA ACAGGCCTCA TCCCACTTCC ACCAGCACCA TAGAAGAATA  1261 ATTCTGGGCA GAAGTCTGTT TTTTTTCATT TTTCCAGGAC AGTTGGATAT TGTCAGGCCA  1321 CTTGTGACCC CAGCCATGTA GTGAGGGTGC TCTTTCTCTG TGCCTGCTCC TTATGAGTGC  1381 AGTGGAAGGA AGCCACACAC TGGTCAGTCA TTTCAGAGGC AGCAGATGCC CAGGGAGACC  1441 CAAGAAAGAG TCAGGTTAGG GAGCAGTGAA AGTGAGGAGG GAAGACAATT CTGTGAACTC  1501 TGTAACTCTT AAAATTTTTG AAAACTCCAT CGTTAAACAA CTTTTAAAAG AAATAACTAA  1561 ATTTTCAAAT GAGTAAGCAG TGCCACCAAC TAGTGTTTTG CCCGATAGAA GAGCCAGCAT  1621 GTTCACGTTA TTTAAATTAG GTGGAAAAAT CTAAACATTT TTATCTTCAT AATTTAAAAA  1681 ATATATATGT ATATATTGCA TATTCACTTT TTCCTTTAGG TAGAGATGAT TTCAATCCAA  1741 ATACTCTTAC TTTAAAAAAT TTCCTTTCCC CAAGAATCTC CTTGGGACTT TGACTTATTT  1801 TTAAAGCTGT GTTGGAGCTC ATCTTGTTCC CTGATGTGTC TCGAGCCCAT TGGTAGGGTC  1861 ATACAAAGCC CACGGTTACA AGCAGTGGTA GGATTGCAGC CGTGGGCCTG CTGGACACAC  1921 ACATACACCA AAGATGTATT TGGATCTGGG CACCCCCTCC CAGGATCCCT GTACTCACGT  1981 GCCAGTCTCC TGACTAGAGC ACTTTACTCT GTTTCCTCAG CCCTGCAGCC CCTGGGAGCA  2041 CACACTGGGT GCAGCCCTGG GCCAGGCACG GGAGGCCCTG CCCTGTGCTG CCCAGGGGCT  2101 GTGTGCACCA CATGAGCACA TTTCCCTCTG GCCTGGCGGC CTCCAGGCTG GCTGTGGAAA  2161 CAGTTCCTGA GGAAATTAGA GATTCTATGA ATTGTAGGAG TATTAAAGAC CAGGCTGTTG  2221 GCACCAGAAC TTAAAGCGAT GACTGGATGT CTCTGTACTG TATGTATCTG GTTATCAAGA  2281 TGCCTCTGTG CAGAAAGTAT GCCTCCCGTG **GGTATACGTT TTTACCTT**TT TTAAAAAACA  2341 TTTTTGTAGA AAAAATAATT AAATCCCCTT TTTGGAAACT TACTGCAGGT TTTGTGCCTT  2401 GACAACCTCT CCCTATGTGA GGTTTGTAAA AAGTGTCCTG TGACTTAACA CAGAAACGCA  2461 ATAAACACAC ACAAAATAGT TTCATGAGTG ATTCTTCAGA TGCCCTTCCC AACTGGTTAG  2521 TTGATCAAGA ATTTTGGGGG TGGGGGTTGC GGAGAAATCA AGTTTAAAAT TCCTTCTGAT  2581 TAAAAAAATA TAGTGGAATA CAATTGTCTG CCGTTTCCCC TTCTTAATGT ATATATTGTG  2641 AGTATTTATT AGATTCGTAG GTCATATTAC TTATCAACTG AGCCAAATGT CTGTGT**GCAA**  2701 **TTGTGTTTCC TTTACCTT**GT AAAATTTTGT ACAGCATAAA TAAGTAAAAA AATCACTGTT  2761 TTTCTCAACT TTTTCAAAAT CAAGGATTGT AAATATTGTA GATTCTTTTT CTGTGTGATG  2821 TGTCCTACTG TTTCATAATG CTGTAACTTG TAGAAATATT GTATATTTAT TTTCTGCTTA  2881 TTTAATGTCT TAATTTCTGA AAAGTATTAA CATCCCTGTC TCCCACTCCC CTGCCGTCCC  2941 ATGAAGTTAA CTCCTGAGAG TTGTCGGGGG TGACTGGAGA GCTCATTGCA GACCACGTGG  3001 TCCTCCAGGG TGGCTCTCCA CCTTCGGGTC CTGGTATTTC CAGTCAAGTG GGTTTCAATT  3061 CTTGGGCTTT GCCGCCCTTA TGATGAAGTG TGTGTTTGAT GCCAGTGAGA AACTCAGTCT  3121 GGCAGGCTAC AAAATTCTAC TCCAAGAAAT ACCCAGCAAC CTTCTGTTTG TTCCAAAGCA  3181 ACTAGCTTAT CATGCAAGCA AATTTTGCTG ACTCCAGGCT TTATCTTTAG GAAAACAAAA  3241 AAACCAAAGT ATTATCAGCA GGTGGGAAAG ATTTTTCTAT TGAAAATTTA TCCCTGACAA  3301 CTCAGCGTTT AGAAAAGAAA TAAAATGTGC CACTTCCAGA GGTGCTGCAT TGCAGTTGTT  3361 CAGGGCTAGG GCCAGGCAGG ACAAGTGAAT GGGTGGGACA GGTGGCTCCT GCCTAAGGAC  3421 CACCTCAGGC CACTAACCCC TTGTGGACAA CTGTGAGTAG CTGGGTTTTC CCCCACCTGC  3481 TGTGCAACTT CCTGTGCTTT GAGGTTGGAC TAACTTGTCT TCAGGAGCTA ATTAACTGTA  3541 CAGCCCTCCC CACGCCCCAC CCATACGGTC ACTGCATTTG GTCAGCCTGC TTCTTCAGGT  3601 CGATGCCCTC CTTCTGATAC TCCATCTCCT TCAGGGGAGG TTGGGGCCCC ACTGGACTGG  3661 GTGTCAAGAT GTGAAAGCTT ATGGGAGCTT TAAGGAGACT TCATGGTGGT TCCATGCAGG  3721 TGGTTCTGCC ATCCCTGCTG ATTTAGCCTG GTGCCTGTGT GTGTCCACTC ACGTACACGT  3781 GGGGTGGGGG AAACGTGTCT ACAGATGACG CTAAATCAGT TGGGGTCTAC TCTAAACAGC  3841 ATTGTGTGTA AGAAGCATCC TCAAGCTCCC AGTTAAGTAA CTTGACTACT TTTATTTGGG  3901 AATTTCAGAC TATAGAAGCT CTCTTATGTT TTATGTCCAG ATTCTGTGAC CACTAGTTAC  3961 TGTATCAGAA CTCATCAGGT ACCCACTTAT AAATAGCACT GATCTGGCTG TATACTGATC  4021 CATCACTAAC CTGTTTTCTA GGACCCAGCG TATGTAGCAT TTGTATTGCA GTTTCCCTGG  4081 CTTACTTGTG TTTTGCACTG ATGAATTTTG ACAGGGTAAT TGCCACTTTA CTTGTGCAAT  4141 ACTGCTGTAA ATAACTGCAG ATTTTTAAAC AATCTTTTAT GTTAATTTTA TAAAAATAAA  4201 ACTTTCAACT AGTTAAAAAA A | |
| 1. *LIFR* 3’UTR sequence with underlined bioinformatically predicted hsa-miR-548ba target sites. TargetScan predicted seed sequence locations are marked in blue: positions 1384-1390 and 5865-5871. With green are market microT CDS v5.0 predicted target sequences locations are marked in green: positions 760-777, 1064-1092, 4378-4397, 5241-5267 and 6366-6391. |  |
| 1 CAGTGTCACC GTGTCACTTC AGTCAGCCAT CTCAATAAGC TCTTACTGCT AGTGTTGCTA  61 CATCAGCACT GGGCATTCTT GGAGGGATCC TGTGAAGTAT TGTTAGGAGG TGAACTTCAC  121 TACATGTTAA GTTACACTGA AAGTGTTCAT GTGCTTTTAA TGTAGTCTAA AAGCCAAAGT  181 ATAGTGACTC AGAATCCTCA ATCCACAAAA CTCAAGATTG GGAGCTCTTT GTGATCAAGC  241 CAAAGAATTC TCATGTACTC TACCTTCAAG AAGCATTTCA AGGCTAATAC CTACTTGTAC  301 GTACATGTAA AACAAATCCC GCCGCAACTG TTTTCTGTTC TGTTGTTTGT GGTTTTCTCA  361 TGTGTATACT TGGTGGAATT GTAAGTGGAT TTGCAGGCCA GGGAGAAAAT GTCCAAGTAA  421 CAGGTGAAGT TTATTTGCCT GACGTTTACT CCTTTCTAGA TGAAAACCAA GCACAGATTT  481 TAAAACTTCT AAGATTATTC TCCTCTATCC ACAGCATTCA CAAAAATTAA TATAATTTTT  541 AATGTAGTGA CAGCGATTTA GTGTTTTGTT TGATAAAGTA TGCTTATTTC TGTGCCTACT  601 GTATAATGGT TATCAAACAG TTGTCTCAGG GGTACAAACT TTGAAAACAA GTGTGACACT  661 GACCAGCCCA AATCATAATC ATGTTTTCTT GCTGTGATAG GTTTTGCAAG CCTTTTCATT  721 ATTTTTTAGC TTTTATGCTT GCTTCCATTA TTTCAGTTG**G TTGCCCTAAT ATTTAAA**ATT  781 TACACTTCTA AGACTAGAGA CCCACATTTT TTAAAAATCA TTTTATTTTG TGATACAGTG  841 ACAGCTTTAT ATGAGCAAAT TCAATATTAT TCATAAGCAT GTAATTCCAG TGACTTACTA  901 TGTGAGATGA CTACTAAGCA ATATCTAGCA GCGTTAGCTG TCCATATAGT TCTGATTGGA  961 TTTCGTTCCT CCTGAGGAGA CCATGCCGTT GAGCTTGGCT ACCCAGGCAG TGGTGATCTT  1021 TGACACCTTC TGGTGGATGT TCCTCCCACT CATGAGTCTT TTC**ATCATGC CACATTATCT**  1081 **GATCCAGTCC TC**ACATTTTT AAATATAAAA CTAAAGAGAG AATGCTTCTT ACAGGAACAG  1141 TTACCCAAGG GCTGTTTCTT AGTAACTGTC ATAAACTGAT CTGAATCCAT GGGCATACCT  1201 GTGTTGCAGG TGCAGCAATT GCTTGGTGAG CTGTGCAGAA TTGATTGCCT TCAGCACAGC  1261 ATCCTCTGCC CACCCTTGTT TCTCATAAGC GATGTCTGGA GTGATTGTGG TTCTTGGAAA  1321 AGCAGAAGGA AAAACTAAAA AGTGTATCTT GTATTTTCCC TGCCCTCAGG TTGCCTATGT  1381 ATT**TTACCTT** TTCATATTTA AGGCAAAAGT ACTTGAAAAT TTTAAGTGTC CGAATAAGAT  1441 ATGTCTTTTT TGTTTGTTTT TTTTGGTTGT TTGTTTGTTT TTTATCATCT GAGATTCTGT  1501 AATGTATTTG CAAATAATGG ATCAATTAAT TTTTTTTGAA GCTCATATTG TATCTTTTTA  1561 AAAACCATGT TGTGGAAAAA AGCCAGAGTG ACAAGTGACA AAATCTATTT AGGTTCTCTG  1621 TGTATGAATC CTGATTTTAA CTGCTAGGAT TCAGCTAAAT TTCTGAGCTT TATGATCTGT  1681 GGAAATTTGG AATGAAATGC AATTCATTTT GTACATACAT AGTATATTAA AACTATATAA  1741 TAGTTCATAG AAATGTTCAG TAATGAAAAA TATATCCAAT CAGAGCCATC CCTAAAGAGT  1801 GTTCTCTTGT CTTCCTTTGT ATCCTCTTTG GCTCCTTCCC TTAGCTTCCA CCCCAGACAT  1861 CAGACCTGGG GCTGCCTTCT CTCCCTGCCA AAGCTTGCTG TTAGCATTGT CTGCTTGCCC  1921 TCAACTCCTG CCTTGTTCCC TGTTGAAATG TATCAATCCC GTACTTTATC TCTCCAGGAC  1981 AAGGATAAGT GTGGCTACAG TAAAAGTTTA TTGCTCTAAA CGGCGCTAAC CTGAGATAAT  2041 GAGAAAAGGC CAGAAGAGAT TGTTTGATCT CTGTCTGTCA ATATATATGC ATTTCAACTG  2101 TGACCATCTG GATCCAGGGC CCTTTCTAAC CTATATAACG ACTCATTTGT TTGGTTAATG  2161 ATGAATATCA CTGGGGCTCT TAAGTCCTTT CGTTCTATCC AAGACTTCAT TGTTTTTTTC  2221 TGTATGAAAA AAGTGCAGAA TATCACTCAC CTTTCCCCTA CTTTTGGATC CCCTACACGA  2281 GGATCACTAG CTTAATGAGA AGCAGAGTAA CGCTATTAAG TCTGGGCAAG CTCCTTATAA  2341 GTTGCTGTGT GCTTAAAAGA ATGTGATTTA TGTCTGGGGC CAAAGCAGTT TTCCTTCTTT  2401 TCCCTCATTG AATTCAAGAC AGCTAGACCC TTGTGTAGAT CCCAGGATAT TACCTCTACT  2461 CCCCCCAACC TGTGTGTGTA GGGAGAGTAA CAACACGGGA AGAGGGGAGG GAAGTTGGAA  2521 GGAGATTGGT CACATGTTCA GTTGTCTGAG ATACTGGTAA ATGTGCGCTG TTTGTCAATG  2581 AAGGTTGCAT GCCTGCATTT ACAAGGTGGT CTCATACTGA TTGTGCTCTG TCCAGTTAGA  2641 TTCGTGGCTT CTCCCTTGGA AGCTACAATT AAGGACTAGT TAGAAGCAGA GGCTAGATGT  2701 GTTCTCTCCA GCCCTTACTA ACTGGGAAAA TAATTGAGAA ATCCCTCTTT TCCTCAGTGA  2761 AAGCCCAGTC ATTGGTTAGA AAAATCTAAT TGTCAAACTA GCATTATGTT CCATCATATC  2821 TGTGGGATAG TAATTCATGT AAGACACATT TTTACCTCTC TTACAAGTCA ATTCACTTAC  2881 AAGCCAGTTT CAAATCAAGC TTCCCTTGCG AGGAATGGAG ATCATTATGG GGCTTATTAT  2941 GACTACATTT CTTTTATGAG TGACCATTTT AAATAATTAA TATTTTATTT AATATTAAAT  3001 TATTAAAGAC TTAGCTAGAC TTTTAACATT AGATGAACCT TGTGAATCAT ATATTGCATA  3061 GATTAGCACA TGTATGCAAT CTAGGTCATC AATTTGTTGA ATTTATACAG CCCTAGATTT  3121 GAGATTGTGG CCCTGAGTAT CTGGATAGTT GAGTTGTGTG TTTATGTGCT TATATGATAG  3181 AGGTGCTTTG TTCCTGCAGC TTAGAAACAG AAGCAGCCAT TCACTGAGAA GCTTAAGAGA  3241 GGAAGTATCA ACCTCATTTT CCAAACCAGC ATTACTTTCA CCGCTCGTGG ATCTGAAAGA  3301 TTACATTTAG AACATTTAGT ACTATTACAC TCTCAGAAAC TGTGGTAAAA AGTCACATTT  3361 TCAAAACTTC ATGCATATTG TATTCTTGTT GGAAATAAGT CCTATAGTTT CTTAATTGTC  3421 TTCATGCCGT CCATATTTAA CAAACATTGC AAGTCCTTTT TATATTTGGG TAATTATTTG  3481 TAATTTAGTG AAGGAGACTA GGGGATGTTT TCTTCCAAAG GGAATTTAAA ATCAATTTTA  3541 TGGTATTTTG AAAGTAAAAT ACTCCTTAAC GTGTCAATAT TTTTAAAATG TTATCTCGAA  3601 GTTTTCTATT GCTCAGGTAT ATAATCTAAT AAAATTTTTT GTTTGCTCTA AGGTTATTTT  3661 TGCTATTCTC TATTTTGAAT TATGTTAAAA TTTGTGTGCA TTTTATGAAA TGCCTTTTTT  3721 CTTATTTTGA AAGTGTAAAA TTGGGCATCT CAGAGTAAAA TGTGGGATTA TTAGCTACAA  3781 AGACTAGGCA CATCGTGAAA GCTCAGTAAC TAATTTTCTT GACTGAGTCC TATCCCAGAG  3841 TGTCACAGAT TCAACAGAAT TTTCCTGAAG GGTGATACAC ACCAAAATCT AGCTTGTGTG  3901 GATCTGAGAC TATTGACTCA AAGATTCAAA TCTACTTCGG AAGTGACTCC ACGGTTTGAG  3961 TGAAAACTGA GGAAGGACTC ATGCCTGTAT ATCACTAGAT ATCTTATTTC TCCATAATTT  4021 TAAGTGTTCA TAATTTCTTC TTCTTTGATG GGTGTGCAAC TGGGGCATGT TTGTACCAGC  4081 CAGCATGTGT TCTGCCCAAC AGTGTGTCCG TGTGTGAGGT TAACTGATTC AGAACTCATT  4141 CTCTTGATAA ACTATTTCTT GAAAGCATTT ATTTTAAGTC TTTATTTCCT GCTGAAATGA  4201 AGTTTCATGC TGTCATGCCC ATCTTATTTA TTCTAGAAGA AAATTTTCAT AGAAGAAAAT  4261 GATTTATGGC CAAAGTTACA GAACATTCAT CTTTTAGACT TAGCCTTAGA ATATCATTGG  4321 AAATTACTTG GCTTGAATTT GGTTCGTTTT TATCTTTATG GATTATGTAC CAGGGCT**ATT**  4381 **AATAATACTT AAACCCC**TTA TTTTGCTATG GTGGTTAGAT TTTTTTATTC CTTTTCAATT  4441 AGTGCTATTA ATTATTACAT GGTCGTATTC ATGCTTATTA CAGATGAACT ATTTTTAGTG  4501 CCAGTGTAAA GGATTAAGAA TAACATTCAA TTAGTTGGCT ATTTCAGGCT TTATTGTACA  4561 TGGTAATGTT TCAGGGAGTG TGATTTGGTC TAGAATATAT CCAGATCCTT GTAATTATGA  4621 TATTGGGTTA TGACAGGATT CTATTATTTT TAGCTATGAA TGGTGCTTAT TTTTCCCTAT  4681 ATTAGGAATG TTAATATTTG GATTATCAGA GTCCATGTTA TAAACAGAAT TTACCTATGC  4741 AAGACAAATT CTAACTGTGC AACATTTTCA TGAGAATTAA AAGTTGTGTG TTAAGGGGAA  4801 AACCCTCTGA ATGTTTTTAT GTGTAAGTGC CTAAAGAACC GTAGAAACAG TACATCAAAA  4861 GCTCTTATAA AAGAAAAACT GATTGAACAT TAGCCAGAAA CAAAATATGA TTTACATTTT  4921 TTTTTTTGGT TGCTGGGACT GAATACCCTA AAAACTAAGA GATGTGAGTG GGGAAAATGT  4981 CTAATTGATA AGAAAGTTGA TTTATATTAT ATTTACATGC ATCTTCTAAG ATTGCCTTTA  5041 CAGTAGCCAA TCAGAACTTT AAAAAAAAAA AAGCTAACAA CATAGATCCT ATGTGTTGCC  5101 CTTAAGACTA AAATACTTTG CACACGTGAT TGAATGGCTC ACTTAAGTTG TTGAATTTCC  5161 TTATACATTT AAAGTGTACT TCCGTTAAGA ATTATTCCAT TTTAAATGGG TAACTTTCAG  5221 CTTCCGAACT ATTTTCCTAA **ACATTCCAGT TGTGTATTAT AAAAGAT**CAA AATATCTCTA  5281 GGTGGTAGTA CAATTTTCAT TTATAACGTG GAAAAGTTAA CTGTTAGGTT TAACATTAGT  5341 ATTAGTGTTT TTGCAATTGC TCATAGAAAA GAACACTGAA TAATGGTTTT CATACAATTT  5401 TTGTAAAACA TTCTTCTCCC AGTTTGATTC CAAATGCTTT TCCCCAAATT GTCTAATATA  5461 TCAT**AAGCTA G**TGTTTCCTT GTATTTAATT TTTCCTTCCA ACACAATATT TAGGAAAAAT  5521 ATGAATAACA TTGTGAGTAG CGGGAGACAA CACGAAAAAG AAACCTTTCA TTGTTGTGGA  5581 GGGGTAGGTG TTATGTGATT GATGCCCTTC CTGGCGTTTG TTTTATCCTG TTATTTTCTC  5641 AAAAAAGAGC ACGGCTTTTT CTTAAATACA TTTAAATTCA GAATTGCTTG CAAAAGATTG  5701 GCCTGGATAG ATAGACATCC TAGATTTAAG ATAGTACCAT CTTAAAAGTA TGTTGAAAGG  5761 TAAAATAAAT CATTTCAAAA TACTTAACTT TCCTAATAAG GATGTTAGGT TTTCTTCTCT  5821 TTCATTAGCT GGCCATAAGT CAAGGTCCTT ATAACACTAG GAAATTACCT TTTTTTACTT  5881 TCAAAATTGC ATTATAGTGA ATTACTGTTT ACAAAATACA CATACTGTGA ACAGATATAA  5941 GTTTCTGTCT TTTATAAGGT TTGTAGAATG AATGTTTTTT TAAAGTGAGT ATTATATGTT  6001 AAATATTTTA AGTTTTTTAT AAATACTAGT AACTGTTTAC TAATTTTTGT TTGGTCAAAT  6061 GCTTGTAAAT GTAGCTGAAA GAAGATAGGG AGAAACTGCG GATCCCAAAC TGTTCCCTTT  6121 TTCATTTCTT GAAATGTTAC CACTACAGAC ATTTTTTTAA GGTGAATAAA CAGTTGTGAT  6181 GTGCTGTACC TAAAATCATG TTTAATCGTA TAAGGAAACA TTTCAATACA CTTATACAGG  6241 AAGAAAACTA TAGATGAAGT ACATGTGTGT GATTCAGTCT GATTCACAGA ATTCTGAGAG  6301 TAATATGGAA TAAAACAACT CCACTTAGAT GATAACTGAA GCATTTCCTG CCTTGTGAAA  6361 AT**TTGGATTT TAAATTGCTG TTAGAATGGG A**AATTTGGAC ACTTTATATC ATTGTATAAT  6421 TTCAGAATTT AGTTTCTGTA TCTTTTGGAA AACATGATTA TAGCAAAAAC ATAGAAAATA  6481 ATCTATTACT AAAACACCAT AAATGTAAAA CTAGTATGCT TGGCTGTTAA CTCTAAAGAT  6541 GTTACTTATG TCTGTTTTTA AAACATGCAT GTATTTAACA ATTTTATCAT AGTATTGTCA  6601 TGGAAAAAAT AAATATATTT TCTTACATCT TA |  |

| 1. *NEO1* 3’UTR sequence with underlined bioinformatically predicted hsa-miR-548ba target sites. microT CDS v5.0 predicted target sequences locations are marked in green: positions 327-346, 390-407, 1982-2007 and 2190-2211. TargetScan and miRDB commonly predicted seed sequence location is marked in red: position 402-408.  \| 1 CGACCTTCAC CAGGACCTGA CTTCAAACCT GAGTCTGGAA GTCTTGGAAC TTACCCTTGA  61 AAACAAGGAA TTGTACAGAG TACGAGAGGA CAGCACTTGA GAACACAGAA TGAGCCAGCA  121 GACTGGCCAG CGCCTCTGTG TAGGGCTGGC TCCAGGCATG GCCACCTGCC TTCCCCTGGT  181 CAGCCTGGAA GAAGCCTGTG TCGAGGCAGC TTCCCTTTGC CTGCTGATAT TCTGCAGGAC  241 TGGGCACCAT GGGCCAAAAT TTTGTGTCCA GGGAAGAGGC GAGAAGTGCA ACCTGCATTT  301 CACTTTGTGG TCAGGCCGTG TCTTTG**TGCT GTGACTGCAT CACCTT**TATG GAGTGTAGAC  361 ATTGGCATTT ATGTACAATT TTATTTGTG**T CTTATTTTAT TTTACCTT**CA AAAACAAAAA  421 CGCCATCCAA AACCAAGGAA GTCCTTGGTG TTCTCCACAA GTGGTTGACA TTTGACTGCT  481 TGTTCCAATT ATGTATGGAA AGTCTTTGAC AGTGTGGGTC GTTCCTGGGG TTGGCTTGTT  541 TTTTGGTTTC ATTTTTATTT TTTAATTCTG AGTCATTGCA TCCTCTACCA GCTGTTAATC  601 CATCACTCTG AGGGGGAGGA AATGTTGCAT TGCTGTTTGT AAGCTTTTTT TATTATTTTT  661 TTATTATAAT TATTAAAGGC CTGACTCTTT CCTCTCATCA CTGTGAGATT ACAGATCTAT  721 TTGAATTGAA TGAAATGTAA CATTGAAAAG ACTTGTTTGT TGCTTTCTGT GCAGTTTCAG  781 TATTGGGGCG GGTGGGGGGC TGGGGGTTGG TAATAGGAAA TGGAGGGGCT GCTGAGGTCC  841 TGTGAATGTT TCTGTCATTG TACTTTCTTC CAGAAGCCTG CAGAGAATGG AAGCATCTTC  901 TTTATTGTCC TTTCCTGGCA TGTCCATCCT TATTGTCACT ACGTTGCAAC TGGAGTTTGA  961 TTTGGATCTG GTTTTAAAAT TCTTCTGTGC AATAGATGGG TTTGAGGATT TAGCGGCCCT  1021 GATGTCTTGG TCATAGCCTG GTAAGAATGT CCATGCTGAG GAGCCAGATG TTGTATTTCT  1081 AACTGCCTGA GTCACACAGA ATAGGGTAAG AGCCTGACCC CATTCTGTAA ATCAGAAAGC  1141 AAGGATGGAG ACCCTTTCCT GCTGCTATTA TTGGCTCTCT TTGAGGAAGT TGGAGGTTAA  1201 GGAAGGAACT TGTTTGTTTC CGTATACGAC TCCTTCTTCT CTCTAGTTCA GTCTTCAGCC  1261 AGTCCAGCGC TCTCTTCCAC ACTTCAGAGC CCCTTCAGAG AAAGCATTAG CAGGAATGAG  1321 ACAAGGCAGA GCTGCAGTGC CCCCTGAGGC TTCCACACAT CTTTCTGAAT ATTATTTTTC  1381 AAGTAACAAG GGCAGGGACA GCGGAAACAG CTGCCCACCC CCCCCATCCC AGCAGCTCAG  1441 CTAAGCCCTG ATGAGAATGA AGCCACAGGA GTTGTCTGAG GTGAACCCAG CCGCTCAGCC  1501 ACACATGGAA GCCATTGCCT TTGCACATAG TTCTTGGGTT CTTTTTCCTA AAAAGGTAAG  1561 GAGCTGAGGT GTGTGGTTTT TTAATATTAA GAATATATAA TGGAAAACAC ACGACTGACG  1621 CTCAGGCATC TTCCCCTACT CCCCAACAGA TCCCCAGAAG ACAGCGTGGA AGGCAGTGTA  1681 GACAGTAAAT CGGGCTTCAG TTCTATAGCC AAGAAGAGAT CAGCTGCTGA AACCACCAGT  1741 GGGTACCCCA GGCCACCTGC CTTTGAACTT GGGGATTTGC CATGTTTGAT CTTGTCACAT  1801 ACTTGCTTTT TTACAAGATG AACTCTTTGT ATTTATGATT TGGGGGGCAA TGAAAGGTGC  1861 AATGCAGGAA CTGCTGCTGC CGAGCTCGCT GGTCACATGG GGGTGCCAGG CGGGATTCTG  1921 GAAAACCAGT GCACTTAAAC TGATCCTGAA GAGAGCTGTC CCAGCACTCT GGCCACCAGG  1981 A**GGGCCAGAT TCCCCAGAAA CTACCTT**TTG CCCAAAGAAC ATGCTCAGTA TTTGGGGCAT  2041 TTCCTCCCAC AAACCCTGAC TGCTTCTGTT ACCTCAGGGC CTTGGTACCT GGATACTGCC  2101 ACAGAATTGG GGCGGGTGGG GGAGGGGCCT ATTTTTAAAT AAAATAACTG TTCAAAGTTG  2161 GGGGTTTTTT AAAAAATTAA GAAAAAGGA**A AGCTATTCTG TATTGCACCT T**TTCACAATT  2221 TAATACATTT TCTTACATTT TCCTGTGATT TTCGAAACTA AACCATTGTG TGTCCTGTAG  2281 TGTCCTGGTT GAGCTGCCGC TCAGCAGCTT CCTCGGGGGG ATTTGGAACA CCTGTGTCTG  2341 TCGCCGCACT GCCTGTGGGA GGGGCCCAGA GGGCTGCTGG GACTGGCGTC TGTACACACT  2401 TGTTTGGCCT TTTCTGTAGT TGATGCTGTA AACTCTATGG CTTTTTAAAA ACGATTTCAT  2461 GTTTTTATTT AGTATTGGAA ATCCAATACA CTTTTTTAAT CCAATCAAAC \| \| --- \|  \| 1. *PTEN* 3’UTR sequence with underlined bioinformatically predicted hsa-miR-548ba target sites. microT CDS v5.0 predicted target sites are marked in green: positions 51-56, 202-224, 566-585, 1890-1906, 2893-2913 and 2986-3006. TargetScan and miRDB common predicted seed sequence location is marked in red: position 2907-2913. For PTEN two isoforms of 3’UTR sequences were used: PTEN short 1-3,300 and PTEN long 1-6,458 nt. \| \| --- \| \| 1 ATTTTTTTTT ATCAAGAGGG ATAAAACACC ATGAAAATAA ACTTGAATAA **ACTGAAA**ATG  61 GACCTTTTTT TTTTTAATGG CAATAGGACA TTGTGTCAGA TTACCAGTTA TAGGAACAAT  121 TCTCTTTTCC TGACCAATCT TGTTTTACCC TATACATCCA CAGGGTTTTG ACACTTGTTG  181 TCCAGTTGAA AAAAGGTTGT G**TAGCTGTGT CATG**TATATA CCTTTTTGTG TCAAAAGGAC  241 ATTTAAAATT CAATTAGGAT TAATAAAGAT GGCACTTTCC CGTTTTATTC CAGTTTTATA  301 AAAAGTGGAG ACAGACTGAT GTGTATACGT AGGAATTTTT TCCTTTTGTG TTCTGTCACC  361 AACTGAAGTG GCTAAAGAGC TTTGTGATAT ACTGGTTCAC ATCCTACCCC TTTGCACTTG  421 TGGCAACAGA TAAGTTTGCA GTTGGCTAAG AGAGGTTTCC GAAGGGTTTT GCTACATTCT  481 AATGCATGTA TTCGGGTTAG GGGAATGGAG GGAATGCTCA GAAAGGAAAT AATTTTATGC  541 TGGACTCTGG ACCATATACC ATCTCC**AGCT ATTTACACAC ACCTT**TCTTT AGCATGCTAC  601 AGTTATTAAT CTGGACATTC GAGGAATTGG CCGCTGTCAC TGCTTGTTGT TTGCGCATTT  661 TTTTTTAAAG CATATTGGTG CTAGAAAAGG CAGCTAAAGG AAGTGAATCT GTATTGGGGT  721 ACAGGAATGA ACCTTCTGCA ACATCTTAAG ATCCACAAAT GAAGGGATAT AAAAATAATG  781 TCATAGGTAA GAAACACAGC AACAATGACT TAACCATATA AATGTGGAGG CTATCAACAA  841 AGAATGGGCT TGAAACATTA TAAAAATTGA CAATGATTTA TTAAATATGT TTTCTCAATT  901 GTAACGACTT CTCCATCTCC TGTGTAATCA AGGCCAGTGC TAAAATTCAG ATGCTGTTAG  961 TACCTACATC AGTCAACAAC TTACACTTAT TTTACTAGTT TTCAATCATA ATACCTGCTG  1021 TGGATGCTTC ATGTGCTGCC TGCAAGCTTC TTTTTTCTCA TTAAATATAA AATATTTTGT  1081 AATGCTGCAC AGAAATTTTC AATTTGAGAT TCTACAGTAA GCGTTTTTTT TCTTTGAAGA  1141 TTTATGATGC ACTTATTCAA TAGCTGTCAG CCGTTCCACC CTTTTGACCT TACACATTCT  1201 ATTACAATGA ATTTTGCAGT TTTGCACATT TTTTAAATGT CATTAACTGT TAGGGAATTT  1261 TACTTGAATA CTGAATACAT ATAATGTTTA TATTAAAAAG GACATTTGTG TTAAAAAGGA  1321 AATTAGAGTT GCAGTAAACT TTCAATGCTG CACACAAAAA AAAGACATTT GATTTTTCAG  1381 TAGAAATTGT CCTACATGTG CTTTATTGAT TTGCTATTGA AAGAATAGGG TTTTTTTTTT  1441 TTTTTTTTTT TTTTTTTTTA AATGTGCAGT GTTGAATCAT TTCTTCATAG TGCTCCCCCG  1501 AGTTGGGACT AGGGCTTCAA TTTCACTTCT TAAAAAAAAT CATCATATAT TTGATATGCC  1561 CAGACTGCAT ACGATTTTAA GCGGAGTACA ACTACTATTG TAAAGCTAAT GTGAAGATAT  1621 TATTAAAAAG GTTTTTTTTT CCAGAAATTT GGTGTCTTCA AATTATACCT TCACCTTGAC  1681 ATTTGAATAT CCAGCCATTT TGTTTCTTAA TGGTATAAAA TTCCATTTTC AATAACTTAT  1741 TGGTGCTGAA ATTGTTCACT AGCTGTGGTC TGACCTAGTT AATTTACAAA TACAGATTGA  1801 ATAGGACCTA CTAGAGCAGC ATTTATAGAG TTTGATGGCA AATAGATTAG GCAGAACTTC  1861 ATCTAAAATA TTCTTAGTAA ATAATGTTGA **CACGTT**TTCC ATACCTTGTC AGTTTCATTC  1921 AACAATTTTT AAATTTTTAA CAAAGCTCTT AGGATTTACA CATTTATATT TAAACATTGA  1981 TATATAGAGT ATTGATTGAT TGCTCATAAG TTAAATTGGT AAAGTTAGAG ACAACTATTC  2041 TAACACCTCA CCATTGAAAT TTATATGCCA CCTTGTCTTT CATAAAAGCT GAAAATTGTT  2101 ACCTAAAATG AAAATCAACT TCATGTTTTG AAGATAGTTA TAAATATTGT TCTTTGTTAC  2161 AATTTCGGGC ACCGCATATT AAAACGTAAC TTTATTGTTC CAATATGTAA CATGGAGGGC  2221 CAGGTCATAA ATAATGACAT TATAATGGGC TTTTGCACTG TTATTATTTT TCCTTTGGAA  2281 TGTGAAGGTC TGAATGAGGG TTTTGATTTT GAATGTTTCA ATGTTTTTGA GAAGCCTTGC  2341 TTACATTTTA TGGTGTAGTC ATTGGAAATG GAAAAATGGC ATTATATATA TTATATATAT  2401 AAATATATAT TATACATACT CTCCTTACTT TATTTCAGTT ACCATCCCCA TAGAATTTGA  2461 CAAGAATTGC TATGACTGAA AGGTTTTCGA GTCCTAATTA AAACTTTATT TATGGCAGTA  2521 TTCATAATTA GCCTGAAATG CATTCTGTAG GTAATCTCTG AGTTTCTGGA ATATTTTCTT  2581 AGACTTTTTG GATGTGCAGC AGCTTACATG TCTGAAGTTA CTTGAAGGCA TCACTTTTAA  2641 GAAAGCTTAC AGTTGGGCCC TGTACCATCC CAAGTCCTTT GTAGCTCCTC TTGAACATGT  2701 TTGCCATACT TTTAAAAGGG TAGTTGAATA AATAGCATCA CCATTCTTTG CTGTGGCACA  2761 GGTTATAAAC TTAAGTGGAG TTTACCGGCA GCATCAAATG TTTCAGCTTT AAAAAATAAA  2821 AGTAGGGTAC AAGTTTAATG TTTAGTTCTA GAAATTTTGT GCAATATGTT CATAACGATG  2881 GCTGTGGTTG CC**ACAAAGTG CCTCGTTTAC CTT**TAAATAC TGTTAATGTG TCATGCATGC  2941 AGATGGAAGG GGTGGAACTG TGCACTAAAG TGGGGGCTTT AACTGT**AGTA TTTGGCAGAG**  3001 **TTGCCT**TCTA CCTGCCAGTT CAAAAGTTCA ACCTGTTTTC ATATAGAATA TATATACTAA  3061 AAAATTTCAG TCTGTTAAAC AGCCTTACTC TGATTCAGCC TCTTCAGATA CTCTTGTGCT  3121 GTGCAGCAGT GGCTCTGTGT GTAAATGCTA TGCACTGAGG ATACACAAAA ATACCAATAT  3181 GATGTGTACA GGATAATGCC TCATCCCAAT CAGATGTCCA TTTGTTATTG TGTTTGTTAA  3241 CAACCCTTTA TCTCTTAGTG TTATAAACTC CACTTAAAAC TGATTAAAGT CTCATTCTTG  **3301** TCATTGTGTG GGTGTTTTAT TAAATGAGAG TTTATAATTC AAATTGCTTA AGTCCATTGA  3361 AGTTTTAATT AATGGGCAGC CAAATGTGAA TACAAAGTTT TCAGTTTTTT TTTTTCCTGC  3421 TGTCCTTCAA AGCCTACTGT TTAAAAAAAA AAAAAAAAAA AAACATGGCC TGAGAGTAGA  3481 GTATCTGTCT ACTCATGTTT AATTAAGGAA AAACACTTAT TTTTAGGGCT TTAGTCATCA  3541 CTTCATAAAT TGTATAAGCA CATTAAATAG CGTTCTAGTC CTGAAAAAGT CCAAGATTCT  3601 TAGAAAATTG TGCATATTTT TATTATGACA GATGTTTGAA GATAATTCCC CAGAATGGAT  3661 TTGATACTTT AGATTTCAAT TTTGTGGCTT TTGTCTATTA TTCTGTACTC TGCCATCAGC  3721 ATATGGAAAG CTTCATTTAC TCATCATGAC TTGTGCCATA TAAAAATTGA TATTTCGGAA  3781 TAGTCTAAAG GACTTTTTGT ACTTGAATTT AATCATGTTG TTTCTAATAT TCTTAAAAGC  3841 TTGAAGACTA AAGCATATCC TTTCAACAAA GCATAGTAAG GTAATAAGAA AGTGTAGTTT  3901 GTACAAGTGT TAAAAAAATA AAGTAGACAA TGTTACAGTG GGACTTATTA TTTCAAGTTT  3961 ACATTTTCTC CATGTAATTT TTTAAAAAGT AAATGAAAAA ATGTGCAATA ATGTAAAATA  4021 TGAAGTGTAT GTGTACACAC ATTTTATTTT TCGGTATCTT GGGTATACGT ATGGTTGAAA  4081 ACTATACTGG AGTCTAAAAG TATTCTAATT TATAAGAAGA CATTTTGGTG ATGTTTGAAA  4141 AATAGAAATG TGCTAGTTTT GTTTTTATAT CATGTCCTTT GTACGTTGTA ATATGAGCTG  4201 GCTTGGTTCA GTAAATGCCA TCACCATTTC CATTGAGAAT TTAAAACTCA CCAGTGTTTA  4261 ATATGCAGGC TTCCAAAGGC TTATGAAAAA AATCAAGACC CTTAAATCTA GTTAATTTGC  4321 TGCTAACATG AAACTCTTTG GTTCTTTTAT TTTTGCCAGA TAATTAGACA CACATCTAAA  4381 GCTTAGTCTT AAATGGCTTA AGTGTAGCTA TTGATTAGTG CTGTTGCTAG TTCAGAAAGA  4441 AATGTTTGTG AATGGAAACA AGAATATTCA GTCCAAACTG TTGTAAGGAC AGTACCTGAA  4501 AACCAGGAAA CAGGATAATG GAAAAAGTCT TTTAAAGATG AAATGTTGGA GCCAACTTTC  4561 TTATAGAATT AATTGTATGT GGCTATAGAA AGCCTAATGA TTGTTGCTTA TTTTTGAGAG  4621 CATATTATTC TTTTATGACC ATAATCTTGC TGTTTTTCCA TCTTCCAAAA GATCTTCCTT  4681 CTAATATGTA TATCAGAATG TGGGTAGCCA GTCAGACAAA TTCATATTGG TTGGTAGCTT  4741 TAAAAAGTTT GTAATGTGAA GACAGGAAAG GACAAAATAG TTTGCTTTGG TGGTAGTACT  4801 CTGGTTGTTA AGCTAGGTAT TTTGAGACTA CTTCCCCATC ACAACAACAA TAAAATAATC  4861 ACTCATAATC CTATCACCTG GAGACATAGC CATCGTTAAT ATGTTAGTGA CTATACAATC  4921 ATGTTTTCTT CTGTATATCC ATGTATATTC TTTAAAAATG AAATTTATAC TGTACCTGAT  4981 CTCAAAGCTT TTTAGCTTAG TATATCTGTC ATGAATTTGT AGGATGTTCC ATTGCATCAG  5041 AAAACGGACA GTGATTTGAT TACTTTCTAA TGCCACAGAT GCAGATTACA TGTAGTTATT  5101 GAGAATCCTT TCGAATTCAG TGGCTTAATC ATGAATGTCT AAATATTGTT GACATTAGGA  5161 TGATACATGT AAATTAAAGT TACATTTGTT TAGCATAGAC AAGCTTAACA TTGTAGATGT  5221 TTCTCTTCAA AAATCATCTT AAACATTTGC ATTTGGAATT GTGTTAAATA GAATGTGTGA  5281 AACACTGTAT TAGTAAACTT CATCACCTTT CTACTTCCTT ATAGTTTGAA CTTTTCAGTT  5341 TTTGTAGTTC CCAAACAGTT GCTCAATTTA GAGCAAATTA ATTTAACACC TGCCAAAAAA  5401 AGGCTGCTGT TGGCTTATCA GTTGTCTTTA AATTCAAATG CTCATGTGAC TTTTATCACA  5461 TCAAAAAATA TTTCATTAAT GATTCACCTT TAGCTCTGAA AATTACCGCG TTTAGTAATT  5521 ATAGTGGGCT TATAAAAACA TGCAACTCTT TTTGATAGTT ATTTGAGAAT TTTGGTGAAA  5581 AATATTTAGC TGAGGGCAGT ATAGAACTTA TAAACCAATA TATTGATATT TTTAAAACAT  5641 TTTTACATAT AAGTAAACTG CCATCTTTGA GCATAACTAC ATTTAAAAAT AAAGCTGCAT  5701 ATTTTTAAAT CAAGTGTTTA ACAAGAATTT ATATTTTTTA TTTTTTAAAA TTAAAAATAA  5761 TTTATATTTC CTCTGTTGCA TGAGGATTCT CATCTGTGCT TATAATGGTT AGAGATTTTA  5821 TTTGTGTGGA ATGAAGTGAG GCTTGTAGTC ATGGTTCTAG TGTTTCAGTT TGCCAAGTCT  5881 GTTTACTGCA GTGAAATTCA TCAAATGTTT CAGTGTGGTT TTCTGTAGCC TATCATTTAC  5941 TGGCTATTTT TTTATGTACA CCTTTAGGAT TTTCTGCCTA CTCTATCCAG TTGTCCAAAT  6001 GATATCCTAC ATTTTACAAA TGCCCTTTCA GTTTCTATTT TCTTTTTCCA TTAAATTGCC  6061 CTCATGTCCT AATGTGCAGT TTGTAAGTGT GTGTGTGTGT GTCTGTGTGT GTGTGAATTT  6121 GATTTTCAAG AGTGCTAGAC TTCCAATTTG AGAGATTAAA TAATTTAATT CAGGCAAACA  6181 TTTTTCATTG GAATTTCACA GTTCATTGTA ATGAAAATGT TAATCCTGGA TGACCTTTGA  6241 CATACAGTAA TGAATCTTGG ATATTAATGA ATTTGTTAGT AGCATCTTGA TGTGTGTTTT  6301 AATGAGTTAT TTTCAAAGTT GTGCATTAAA CCAAAGTTGG CATACTGGAA GTGTTTATAT  6361 CAAGTTCCAT TTGGCTACTG ATGGACAAAA AATAGAAATG CCTTCCTATG GAGAGTATTT  6421 TTCCTTTAAA AAATTAAAAA GGTTAATTAT TTTGACTA \| \| 1. *RARB* 3’UTR sequence with underlined bioinformatically predicted hsa-miR-548ba target sites.   microT CDS v5.0 predicted target sequences locations are marked in green: positions 816-835  and 1257-1270. TargetScan and miRDB predicted seed sequence location is marked in red:  position 1264-1270. \| \| \| 1 GACATTTTCT AGCTACTTCA AACATTCCCC AGTACCTTCA GTTCCAGGAT TTAAAATGCA  61 AGAAAAAACA TTTTTACTGC TGCTTAGTTT TTGGACTGAA AAGATATTAA AACTCAAGAA  121 GGACCAAGAA GTTTTCATAT GTATCAATAT ATATACTCCT CACTGTGTAA CTTACCTAGA  181 AATACAAACT TTTCCAATTT TAAAAAATCA GCCATTTCAT GCAACCAGAA ACTAGTTAAA  241 AGCTTCTATT TTCCTCTTTG AACACTCAAG ATTGCATGGC AAAGACCCAG TCAAAATGAT  301 TTACCCCTGG TTAAGTTTCT GAAGACTTTG TACATACAGA AGTATGGCTC TGTTCTTTCT  361 ATACTGTATG TTTGGTGCTT TCCTTTTGTC TTGCATACTC AAAATAACCA TGACACCAAG  421 GTTATGAAAT AGACTACTGT ACACGTCTAC CTAGGTTCAA AAAGATAACT GTCTTGCTTT  481 CATGGAATAG TCAAGACATC AAGGTAAGGA AACAGGACTA TTGACAGGAC TATTGTACAG  541 TATGACAAGA TAAGGCTGAA GATATTCTAC TTTAGTTAGT ATGGAAGCTT GTCTTTGCTC  601 TTTCTGATGC TCTCAAACTG CATCTTTTAT TTCATGTTGC CCAGTAAAAG TATACAAATT  661 CCCTGCACTA GCAGAAGAGA ATTCTGTATC AGTGTAACTG CCAGTTCAGT TAATCAAATG  721 TCATTTGTTC AATTGTTAAT GTCACTTTAA ATTAAAAGTG GTTTATTACT TGTTTAATGA  781 CATAACTACA CAGTTAGTTA AAAAAAATTT TTTTAC**AGTA ATGATAGCCT CCAAG**GCAGA  841 AACACTTTTC AGTGTTAAGT TTTTGTTTAC TTGTTCACAA GCCATTAGGG AAATTTCATG  901 GGATAATTAG CAGGCTGGTC TACCACCTGG ACCATGTAAC TCTAGTGTCC TTCCTGATTC  961 ATGCCTGATA TTGGGATTTT TTTTTCCAGC CTTCTTGATG CCAAGGGGCT AATTAATATT  1021 AACAACTCCC AAAGAAACAG GCATAGAATC TGCCTCCTTT GACCTTGTTC AATCACTATG  1081 AAGCAGAGTG AAAGCTGTGG TAGAGTGGTT AACAGATACA AGTGTCAGTT TCTTAGTTCT  1141 CATTTAAGCA CTAGTGGAAT TTTTTTTTTT TGATATATTA GCAAGTCTGT GATGTACTTT  1201 CACTGGCTCT GTTTGTACAT TGAGATTGTT TGTTTAACAA TGCTTTCTAT GTTCATA**TAC**  1261 **TGTTTACCTT** TTTCCATGGA GTCTCCTGGC AAAGAATAAA ATATATTTAT TTTAAAAAAA  1321 AAAAAAAAAA AAAAAAAAAA AAAAAAAAAA AAAAAAAAAA AAAAAAAAAA AAAAAAAAAA  1381 AAAAAAAAAA AAAAAAAAAA AAAAAA \| \|  1. *SP110* 3’UTR sequence with hsa-miR-548ba target site at position 33-40 predicted by microT CDS v5.0 and marked in green. |
| --- | --- | --- | --- | --- | --- | --- | --- |
| 1 ATTTCTACTA CCCTCTCAGT CACCATGTTG CA**GACTTTCC** CTGTCTGGAG GCTCACCTTA  61 GAGCTTCTGA GTTTCCAAGC TCTGAGTCAC CTCCACATTT GGGCATGGCA TCTTCAAAAC  121 AATTAATTTG CATAGTTAAT TTGGGATGGG GAAGCAAATG ACTCTAAAAT AAAAATTAAA  181 TGAAAAAGCT CAAAAAAAAA AAAAAAAAAA A |

| 1. *ADAM19* 3’UTR sequence with hsa-miR-7973 target sequence commonly predicted by TargetScan and miRDB is marked in red at position 624-630. |
| --- |
| 1 ACCTGTCCAA GGGGCTTCTC CCTTTCCTTG AGCTCTCTGG ACACTGCAGA GGACCCATGG  61 CCATGGAACC CTGAAGAAGC ATGTCTGGCC GCCTCTGAGC TCCTCCCACC CTCCTCCAGG  121 AACCTCCACA TCTCCAAAAA TCTCCCTGTT GACTCAGTGC CTCCTCGGCT TCCTTGGAAG  181 CCCAGAGGGA CTATGATCTG ATGGCCTCTA GGTGTTGTTT TGTGCAATAT ACAGCCCCAG  241 GTAGGGAGGG GAGAGTATGA GGAGGGTGAC TGGCAGCTTC TCCTCCAGAC TCCTAGCCCC  301 GAGGTGCTGA TGGAGATGCT CAAGGCCAGC AAGCCCCTCA GGCCAGCACT TCGCTTGCAG  361 AAGCCATCCA TTCACTCCTG GGGTGCAGGG CACGCAAGAG AGCTTCCCAT TGCTTCTGCT  421 CTCCTCAGAG GTCCCGGGCT GGATGGAGGC TGGTACTTAC CCACCCCTTT TAGCTTTTAG  481 GGATTAAGGA AGGGTCAAGC CAGCCACTGC TGTGGCCCTG CCCAGGGCTT GGTTGAGGGA  541 ACGGCTTCTG GCTGTATGGC TGCATGTGAC AAGCCACGTC CCCTCCCACC TCTCCCCAAA  601 CCCCTGCATC CCTGTATTCA CAC**GGGTCAC** TCTGACTCAG ACAGGTACTA TTCGTAGGCA  661 GTGTAGACAG CAGGAGGAGC ACCGGGCTTG GGCTTCCTCT GAGCCGTGAT GCCAAAGGTT  721 GCGACTCCTG ACTCTGGATA ATTTTTAGTT GCTCTTTGTT TTCTCTGCCG CACTTTCCTG  781 GTGCCCCACG CTTTTCTCTC TTCCTTCCCC TCTCATTCTC CCTCTAATGT GTGGTGCTTT  841 GGTGAGCAAA CCCTCAGCAG TCCTGACCTT CGGGTGACCA GGTGCTTGTG ACCTACAAGT  901 CAGAGTCCTC TCTCACAGTC GGCCACTGGA TTTCCCTCAC TGGCTCTCAG GAGTGTGACC  961 AGAGTAGACT TGGGGCATGG CCATTGGGGT CATATGTTTA TTTTTCATTG TGTTTTGTGA  1021 CCTCAGCAGG GTGGGGGTCT TCCTCCTTAC TCTAAGCTAA ATCTAGGTGA GGTTTCCCCT  1081 TAGGGAGCCC AGCTATTTAC AAAGTACACA CGAGGGAGCA GGCTGGTCAT TGACTTCGGG  1141 CTGGACCGTT GCCCTCTGAG CAGAGAACAG ACCCATTTCT GGGAGCTGCC CGAGATCACT  1201 GGAGAAGGCA GCCAGCAGCA GCTGCACTGG AACAGTCAGA GCAGGGAGCC TCTTCCTCAA  1261 CCCAGCTTTT TGTCATTCAC TTCCTTTTGT TCTCTCTCTG GTCACTGCCC TTACCTGACC  1321 CTCACAGAAA GAGAGCTCTG AGCAGGTGAG GGGGTCTGCG GTGGCTCCTG TCTTCCCTGC  1381 AGCAGGGAAG GAGGGCCGTG TGGTGCTTTG CTAGATAGGA CGGTTTTTGC AAAGCACCTG  1441 GAGATGTTTG CTGGGAGATA GACTCCCACT CCACAAAGGT GCTGGGTGGC TCTCCGGACA  1501 GGAGCTGGCC TGACTCTCAC TCCTCTGAGG CTTTCCTGGG GCCTCCTCCC ATCCTGCCAT  1561 GAGCAATTGT TTGCTCTTGA AAACCTCACT GCAAGGCTGA GGCTGAGCTT CTGATTCACC  1621 ACCCCAGGGC CTCCTTATAG TTCTCTGCAC ACAATAGGTG CTTCTTGGAT GTTCTTGGGT  1681 TTGGAAATAA GTGGAAAATA CGGGATGTAC CCCTGGGGGA AAAGCCTGGG TTGGGTTTAG  1741 AAAGATCTCA GGAAAATGAG TTTCTCTTCC CTCAGGGTGG CTGTGATACA GGTTCCCCAT  1801 GTCCTTGCCG TGGGTCATCC TTGCTGTGGG TCATCCTTGC TGTGGAGATC CATTCCCCAC  1861 CTTTCCTGTG GCCCAACCTT TTATTTAAAT GTGCTACCCT CTGCCTCAAG GCTTGGTTCC  1921 TGGAAAGTAA AGGTGAAAAC ATCCCCTTTC ACCCCTCTGC AAAACAAACA AGCAACATCC  1981 TCAAAACCCA ACCCCATGCC TCACAGAGCT TCCTGTGGCT TCTCCAGCCT TTCTCCCTCA  2041 CATCAGGAGG TAGATAGCTC TGAAATGACA GCGCCACAGC CATAGTGACT GCATGAGCCA  2101 TCTGAACCTG CAGTCCACCC TCCCTGGAAC CACACCAGAA AGAGACCTGG GTTGTCGTTT  2161 TCTTGCTTTT TGTTTTGTTT TGTTTTATTA TTTTCATATC ACCTCCATCC CATAAAGTTG  2221 TACTGTGAAC TGGAAGATGG TGGAATGTTT TGGAATTTGA TAGACTTTCG GCAACCAGTT  2281 CTACTAATGC TTCACTCCTG GCTCTGTTCA GGGAGGCTGC CCAGGAGGAA GACTGGCCAT  2341 TATGCATCCC CTTTTCTTTC CAGTGCCCAG TATGCTGTTT TGAGGTGTCA AATACAAATA  2401 AATCTGGGCT TAGGGAAGGA GAGACCTTAT TCCAAAGCAC GATTGCAGAA GGGGAAAGGG  2461 AATATTGCAA AAGGGAGAGG AAGGGGCCTT ATGGGAATAG TGAAAAGGCT CAGACCGACC  2521 GATGGCAAGA TCTGCAAGCG TCTCAAAGCC CAGGCAGAAA AGGACTTTTC TTTTATTGGA  2581 AGAAGTAAAC ATGGCTAGAA AGAACCACGT TCAGGGAATG ACGTTGTGCC CAGCCTTTTT  2641 TTTTTTTTTT TTTTTTTTGT CTCCAGGGGA GGGGCTGTTT GCTGGCTCAG GCTGAGGATG  2701 GCCCAAAGTC CAGGGTCTGG TGGGGAGGAG GGAAGCTTAA CTCAAGTTTG GGTTAGTGAG  2761 TTAGCAAGCT CTTTGTGCAG ATGGGGATGT AGGTAAATCT TTTTAAAAGT GAAATTAACC  2821 TCCTGCCAAT TTTACAACCC AAGAATTTTT TTTTAAGGGC CTTGGAGCCA TCTCTAAAAC  2881 AAACCTCAAG GGATTTAGTG CCCTGTCTCC CTGTCTCTAG AAGCCTTAGC CTGGGCACCT  2941 GGCTCAATCT TGTAACTGCC TGCTAGCCAT AGATTCCTTT CAGCCTTGCT GACTTCTCCC  3001 TATAAAAGTA AAGCCTTTTT CTGCCCCAGC TCTGAGACAC TTGCAGATCT TAAGGTCTGA  3061 GACTTGCTGA TTTTCTGGTT GGAGTGTTTT TTTGTATTGC CATAGTCCCT TCCCCCTGAA  3121 GCAATAGCCC CTCCCCACCT CCTGCAATAC GCCTTTCCAA TCTTTATTGG AAGTCTCTCC  3181 CTGCCTACTT CCTAATTTAT TCTTATTTGA CAGAGGGTAT GGAAGACTTG CAATTTGAAA  3241 ACTGGGGACC AGTTCCAAAG TCAGTAATTG TGTTAACCAC GTGTATAACA GCTCTGCTGG  3301 ACACCCAAGA AAGCCATGGG AACGCCAACT GGAAAGGTCC CCTTCCCCAG GGGAGCCTGC  3361 GAAGGAGAGG TTCTGTAGAA TCCAAGCCCA CATTTCCAAA GTCACCCCCA ACGCGTCCTC  3421 TCACACCGTC CACTGTGCGT TTGTATGTGT CTGGGATCCA GGGCAATGTG AATTTTCTTT  3481 TTATTTGGGA GATTGTTCAC GGAAAACAGA TCTTCTTCTC TCTTGTCCAC CTATTAATTG  3541 TTTACAATAT TTGTACATCT ATGCAAAATA CTTGAATGGG CCATGGTGCC TTTTTTCCTT  3601 GTTAGTATTT AATTAAAAAT GAATTGTTTG TCATTTGCAA TGTTAAAAAA AAAAAAAAAA  3661 AAAAAAAAAA   1. *ATHL1* 3’UTR sequence with hsa-miR-7973 target sequence predicted by TargetScan is marked in blue at position 346-352.   1 TCAGGAACGG TGGCTTCAGA GACGTCTCTT GGGCCTTCCC TCTGGCCACG TCTGCACCCA  61 CCCCTCCTGG GCACCCTCCT AGCCTGCCAT CCCTCACCTG CAGCCAGGCT CTCAGGGAAG  121 GTCCATGCTG CTTGGCCTGA GTTCAAGGCT TTCTGCCTGT AGCCTGGACT CCCGTGGACC  181 CCCGTGGGCA GGTGGCTTCC CCGTGGCATC TCCACACCGC CTCTGCCTGC CCCTGTGGAC  241 TGATGCTATC GCGCACCGTC CCACGACCCC ACCCCGAGCT CCTGAAGCCG GGGTCTGAGC  301 CTGCATCACC TCTGGCCTCT CATCCCCCAC TCTCCTGAGA GCAGT**GGTCA CA**GCGGCCGG  361 CCGCTCTGCT GAGAAGGCAG AGAGGCAGGC TCAGGCCTCA GCGTGGACAG CAGGGATAAG  421 GGGCACGAAG GACGGGGACT CGGCCCCTTC AGAATTCCTC AGGACTCTCA GGTGCAGCTT  481 TGCCAAAAAG GAACTTTTCA TGTCATGCAG TTGAGGGGAC TTAGTCTCAA TCCCAGGCTC  541 CTCTTGACTC TGGGCAGCTT TAATCAGGTT GGGCAGCCTC TGCTACAGCG TGGGGTGGGA  601 TGGCTCTCTT CCCTCAGCCA CGCCGCTTGT GAGGACAGAG GTGGGGGAGT GGGAAGTGGG  661 AAGTCACCAG AGAACAGGAG AGGGATTTGA GGGCGAGACC CCAGCGCTCT CCACGGACCA  721 GCCAGAGGGA CTGGAGCCAG GTGTGCATGG GTTCAAGGCC CTGGCCCTGC CCAGCCTTTG  781 TCTTGGGAGC TCAGCCCCAG GGTTCGGTCG TCAGCAGTTT CCCAAGAACA AGATGTGATG  841 GCATCTGCTG CTGAAACCCT GATGAGGACC AGGCCCCCTG CACCGCTGTC AGCCTGAGGA  901 ATTAAAGCTT TGGTGCTGGG GAGAGCATTA TTCCTCTGAA A |

| 1. *ATP6V1A* 3’UTR sequence with underlined bioinformatically predicted hsa-miR-7973 target sequences. microT CDS v5.0 predicted target sequence locations are marked in green: positions 1336-1352, 1543-1553 and 2307-2323. TargetScan and miRDB commonly predicted target seed sequence locations are marked in red: position 1547-1554. |
| --- |
| 1 AAGCCTTGAA GATTACAACT GTGATTTCCT TTTCCTCAGC AAGCTCCTAT GTGTATATTT  61 TCCTGAATTT CTCATCTCAA ACCCTTTGCT TCTTTATTGT GCAGCTTTGA GACTAGTGCC  121 TATGTGTGTT ATTTGTTTCC CTGTTTTTTT GGTAGGTCTT ATATAAAACA AACATTCCTT  181 TGTTCTAGTG TTGTGAAGGG CCTCCCTCTT CCTTTATCTG AAGTGGTGAA TATAGTAAAT  241 ATACATTCTG GTTACACTAC TGTAAACTTG TATGTAGGGT GATGACCCTC TTTGTCCTAG  301 GTGTACCCTT TCCTCATCTC TATTAAATTG TAAACAGGAC TACTGCATGT ACTCTCTTTG  361 CAGTGAATTT GGAATGGAAG GCCAGGTTTC TATAACTTTT GAACAGGTAC TTTGTGAAAT  421 GACTCAATTT CTATTGTGGT AAGCTCATTG GCAGCTTAGC ATTTTGCAAA GGAATTGCTT  481 TGCAGGAAAT ATTTAATTTT CAAAAACATA ATGATTAATG TTCCAATTAT GCATCACTTC  541 CCCCAGTATA AATCAGGAAT GTTTGTGAGA AACCATTGGG AACTATACTC TTTTTATTTT  601 TATTTTTTAT TTTTTTTATT ATTTTTTTTT TGGGGACGGA GTGTCCCTCT TGTTGCCCAG  661 GCTGGAGTGC AATGGCGTGA TCTTGGCTCA CTGCAGCCTT CGCCTCCCGG GTTCAAGTGA  721 TTCTCCTGCC TCAGCCTCCC GAGTAGCTGG GATTACAGGC ATGCTCCACC ATGCCCAGCT  781 AATTTTGTAT TTTTAGTAGA AACGGGGTTT CACCATATTG GTCAGGCTGG TCTCGAACTC  841 CAGACCTCAG GTGATCCGCC CACCTCGGCC TCCCAAACTG CTGGGATTAC AGGCGTGAGC  901 CACCGCGCCT GGCCAGGGAC TATACTCTTT TTAAAATAGA CATTTGTGGG GCTCACACAA  961 TATATGAAAT AGTACCCTCT AAAAAAGAGA AAAAAAAAAT CAGGCGGTCA AACTTAGAGC  1021 AACATTGTCT TATTAAAGCA TAGTTTATTT CACTAGAAAA AATTTAATAT CAAGGACTAT  1081 TACATACTTC ATTACTAGGA AGTTCTTTTT AAAATGACAC TTAAAACAAT CACTGAAAAC  1141 TTGATCCACA TCACACCCTG TTTATTTTCC TTAAACATCT TGGAAGCCTA AGCTTCTGAG  1201 AATCATGTGG CAAGTGTGAT GGGCAGTAAA ATACCAGAGA AGATGTTTAG TAGCAATTAA  1261 AGGCTGTTTG CACCTTTAAG GACCAGCTGG GCTGTAGTGA TTCCTGGGGC CAGAGTGGCA  1321 TTATGTTTTT ACAAA**ATAAT GACATATGTC AC**ATGTTTGC ATGTTTGTTT GCTTGTTGAA  1381 TTTTTGAACA GCCAGTTGAC CAATCATAGA AAGTATTACT TTCTTTCATA TGGTTTTTGG  1441 TTCACTGGCT TAAGAGGTTT CTCAGAATAT CTATGGCCAC AGCAGCATAC CAGTTTCCAT  1501 CCTAATAGGA ATGAAATTAA TTTTGTATCT ACTGATAACA GA**ATCTGGGT CAC**ATGAAAA  1561 AAAATCATTT TATCCGTCTT TTAAGTATAT GTTTAAAATA ATAATTTATG TGTCTGCATA  1621 TTGCAGAACA GCTCTGAGAG CAACAGTTTC CCATTAACTC TTTCTGACCA ATAGTGCTGG  1681 CACCGTTGCT TCCTCTTTGG GAAGAGGAAA GGGTGTGTGA ACATGGCTAA CAATCTTCAA  1741 ATACCCAAAT TGTGATAGCA TAAATAAAGT ATTTATTTTA TGCCTCAGTA TATTATTATT  1801 TAATTTTTTA GGTAATGCCT ATCTCTTGGT CTATTAAGGA AAGAAGCAAT CAGTAGAGAA  1861 TTCAGGATAG TTTTGTTTAA ATTCTTGCAG ATTACATGTT TTTACAGTGG CCTGCTATTG  1921 AGGAAAGGTA TTCTTCTATA CAACTTGTTT TAACCTTTGA GAACATTGAC AGAAATTATG  1981 CAATGGTTTG TTGAGATACG GACTTGATGG TGCTGTTTAA TCAGTTTGCT TCCAAAGTGG  2041 CCTACTCAAG AGGCCCTAAG ACTGGTAGAA ATTAAAAGGA TTTCAAAAAC TTTCTATTCC  2101 TTTCTTAAAC CTACCAGCAA ACTAGGATTG TGATAGCAAT GAATGGTATG ATGAAGAAAG  2161 TTTGACCAAA TTTGTTTTTT TGTTGTTGTT GTTGTTTTGA ATTTGAAATC ATTCTTATTC  2221 CCTTTAAGAA TGTTTATGTA TGAGTGTGAA GATGCTAGCG AACCTATGCT CAGATATTCA  2281 TCGTAAGTCT CCCTTCACCT GTTACAG**AGT TTCAGATCGG TCA**CTGATAG TATGTATTTC  2341 TTTAGTAAGA ATGTGTTAAA ATTACAATGA TCTTTTAAAA AGATGATGCA GTTCTGTATT  2401 TATTGTGCTG TGTCTGGTCC TAAGTGGAGC CAATTAAACA AGTTTCATAT GTATTTTTCC  2461 AGTGTTGAAT CTCACACACT GTACTTTGAA AATTTCCTTC CATCCTGAAT AACGAATAGA  2521 AGAGGCCATA TATATTGCCT CCTTATCCTT GAGATTTCAC TACCTTTATG TTAAAAGTTG  2581 TGTATAATTG TTAAAATCTG TGAAAGAATA AAAAGTGGAT TTAAATTAAC AAAAAAAAAA  2641 AAAAA |

| \| \| 1. *FMNL3* 3’UTR sequence with bioinformatically predicted hsa-miR-7973 target sequences.   microT CDS v5.0 predicted target sequence locations are marked in green: positions 805-824,  2481-2493, 3760-3772, 3894-3903, 4262-4269, 4657-4682, 6248-6260, 7301-7314 and 7635-  7642. TargetScan predicted seed sequence locations are marked in blue: positions 233-239,  1620-1626, 3606-3612, 3974-3980, 5387-5393 and 7063-7069. \| \| --- \| \| 1 GACCCTCTCG GAGGCAGAAG CACTTCACCC CTCAGAGTCC TACAAGTCCA ACCAGTGGAC  61 CTGGAATTGG CCAAGGGCTC AGGAGAGGGC TGTGTTGCTC TCTCAACCAT GTCCGCCCCA  121 GCTCTTGAGG CTGGATCTTT CTACTTGTGC CACTATGGGC ACTAGGTCTG TAGGTCCCTG  181 GTGCTTCCAG TACTGCACTT TGCCCCCAAG GTCTTTGACT TCATTTCCTG AA**GGGTCAC**C  241 AAGGGTCAGC TCAGGGCTAG GAAAAACCAT TTCCAGCCTT AGCCTAAACA GGGCACATAG  301 ATCTCTCCCA CTAGAGCCCA GAGGGATGAG AGGAGAGAGC AACTGTTCTT TCTTTTTTTT  361 TCCCTTCCTT TTTGCAGGCC CATGGTCAGC TCTGGTCTAG ATGTATCTAG GCTTAGGGAG  421 CCCTGGGACC CTGCCAGTCT CAGTTCATCT CTTACCAGGA GCAAGGCCCT CTGAAGCATG  481 GGCCGTGCCA GCTGTGCCCT AGTGATAAGG GTAAGAGGAG AGATGTCAGG CTCACTGCCC  541 TTATTTCTCT GCCCATTTCA TTCTAGGCTC ACACTGTCCT TGTCAAGGTG GGCAGAAGAG  601 CAGAGGTCTT CTCTGCACCA ACCACTCTCT GCAGATGGCT GGAGAAAGGG TGTGCCAACT  661 CTTCACCTTC TTTCCTCAGC TACGGTTTTT TTTTTTTTTT TTTTTTTTTT GGCAGGGGAC  721 AAGGAGCATG GTGGTCTTGG CTATTTGCTT ACCTTCCCGT TTCTCCTCTG CCCCTGGAAG  781 GGAATGTGGG GGCCCACTTT TTTG**TACATG TACCACCTCC CTTT**CCTCTT ACTGTACATA  841 AACCTCAGAC TCTCCCCCTC TCAACAAAGG CTTGATGCAC CAGGCCTGAC TTTGTCTCCT  901 CTTTCTGAGC TTAGGGGGCT AGGGTGGCCA TTTAGTCTGA CCTGGGCTAT GGGGATAGAA  961 AAGAAATCTT TGGGGCACTG CATCTGTTTT GGAGGAGATG AGATCAGAAC TCTGGGGAAA  1021 GGAGAACCGG CTAGGTGGGC TCTAGCCACA CAAAGGGAAG CAGGCCTGCC ATGAGACACT  1081 AGTGCCCTCT GCTGGGCATG TGTCCAGTCC CCAGCCTGCC CCAGTAGCAC ATGGAAAAGA  1141 GTCACTGGCC ATGTTATAAA TATTGTTATT TAAAAAACAA AAAACAAAAC ACACGTACAT  1201 TAGGTCCTGG GTGGAGGAAG AAGTGGTGGA GCCTCAGGGC CAGGGCAGGC AGGAGCAGGC  1261 ACTGATGCTA GAGGGTGATG ACCCTGCCCT CTGCCACCCA GGCTGCGTCC AACAGCCTGT  1321 GACTCTTCCC TGGAGACCCC TTCTCTCACA CTTTTATCTA CTGGCATCAC CCACCCCACA  1381 TGGCATATAC CACCCATCTC TGGTCTGGGG CTGAGGGCAA GGACTGCACT AGTCCAGGGA  1441 GTAGGGGACC CTTGAACACC AAGAGCAACC TTGAGAGGTG CCAACAATGG TGTGCTGGGG  1501 GTGGGGTTAC TTTAGAAAAA GTGGACACAG ACCAGACTAA GGAAGAAGTC TGAGGCAGAC  1561 GGTGAGGGTG GCCCAGGAGC CATGGCCTCA CACAGACCCG AGGCAGAGAA CAGCTCATT**G**  1621 **GGTCAC**TGGT GATCATCCAG CTGCTGTAGG AGTGTCCGCC GCCGCCTCTC CAGCTCACCC  1681 TCACTCAGCT CACTTTCTGA CGTGTCCCAG CCTGTCTGTT GGAGCAAAGG CACAGTTCAC  1741 CCTTAGCCTG CACTAAGGGC CGTGGAAGCT TGACCAGCGT GCTGACTGGC CTAGGGCCCC  1801 TGCCCCTCAC CTTCTCCTTC TTGATTCCAA AGCCTGGGGA ACGGTTAGGG AGCTCTGCCT  1861 GTTGGAGCTC CCTGTCCTTG TCCTGTTCTT GTTCTTTCTC ATCGCTCTCC TTGCCAGCTT  1921 TCTCCTCAGG GTCTGTCTCA CTCTCAGGAC TATTCTGTAA GAGTCCAGAG CCCACCTCCT  1981 CAGTTCCTGT GGATAGCCTG GGAGGCAGGT AGAACAGAAC TCACAAAATA GCAGGGGCTT  2041 AGGTAGAAGT TCCTTCACTC ACCGACTTGT GTCTTCTCTT CTTAGTTTTC TTTTTTGGTT  2101 TCTTGGCTTT CCGAAGGCCA TGATCTAGAG ATAGAAAAAT CCCTACTAAG TCCCAACAGG  2161 AGAAAGACAG GCTCTTTTTC TTTGTTCTAG CCCCTCTGTT CATTCTTCAC AGGCAGGAAC  2221 CTCATGCTCT GAGGAAGTCT AAGTCCAGTT TTCCTTCTCC AGCTTCTTGC TTGGGCAGGT  2281 AGAGGAAGTT CTTTCTACTT CCACAGGTGC AGCCCTCCCA ATCCAGTCTA CCCTCAAAGG  2341 AGCTGGGGCT TGTGGAGGGC ATCCTCTCAG TCTGGGACAA CAGTTCTCCA GCTTCTACGC  2401 TCACAGCAAC TGCTTACCTG CTCCAAGAAG ATGGGAGGAA GGGGAGCCCC GTCCTCCAAG  2461 GGCAGCACCC CCACTTTCAA **CTGAATCAAG TGA**GGAAGAG GGCTCAGAGC CTGACTCTGA  2521 GGGGTTCCGC CTCCTCCGCT TGGGGGGCCG GAGAGATGGT GGGGGCAGCT CCTCTTCTTC  2581 TGACTCAGAG CCCTGGGCAG ATCAGCAGAT ACATGTCAGT GCAGAGGTTC TCTGCCCTCC  2641 AGAAGCCCTC GTCTGGGAAT TCGAGAGAGG AGGAGGACCC AGGGCCAAAG GAATCCTCAA  2701 ACTTCCCTCC AAGGGAGATA TTGTAAGGGG AAAACAAAGT GTTTATTTCC CCAAAACAAC  2761 CCAGCTTCAC CCCATGTCCC TTCTACACGC TTACTCACTG AGGGTGAGTG GGAACGCTTG  2821 TGATGGTGCT TCTTGCCTTT CCTGCCATGC TTTCGGCCTT TGGTGTGGAG GTGCTGGCAT  2881 TCAGTCTGCT AGAGGCAGAG GAAGAAGGGT GAGGACAGCA CCAGACTTAG CCCCAGGAAG  2941 GATCCTACTT CCTAAAATCT GGCTTTGCCT CCCTTTCTCA GACTAGAGCC TTCTGCCTAT  3001 CCCAGGGATC CTCAGGCCTC CCCTTCCTTA GCTGGCCAGG AGTGCCCTAA GGGTAAGAGG  3061 GAGGCCACTG AGGTCTCTGT TGAACTGGTC TAATGAGTTC AAGGGCCTGA GGCAGATCCA  3121 GAGGACAAGC CTGCCTCACC TCCAGCACCT GTAGGAACTC CCGGAAGAGC CGGATCCGCT  3181 CCGACTCCAG GGTGATCTGC TCAAAGGCTG AGTCACACAC AAAACGCTCA CGGACCTTGA  3241 ACGAGGAGAG CACTGCTGAT CAGACTAGGC CCCACTCAGC CTGGCCTGGA CACCGCCATG  3301 AGCACAGGGC AGGGCTGGGG CAGAGAAAGG AAGGGAACTG GAGCTGGCCC AGGGCTGTGA  3361 CGTGACTGGG GTGGGCCCTC AGTGGTGAAG CACCGCTGCC CTCAGGCTTG AGGGGTGCTT  3421 GGGGCCAGGC TACGCTCCTG ACCTCTTCCC AGGCAGTGCC TAGCTCCAGA GCAGGCACAG  3481 CCTGCCTCAG CATGCTTCGA AAGGCAGCTT CCCTGCGCCG CATCCTGCGT GCCTCCTCCT  3541 TCTCCCGCTC CCTCTCCCGT GCCTCTGCTT TCTCCAGCAG CTGAGGAAAG AAGGGACACG  3601 GAACA**GGGTC AC**CCAGGAGG GTGGATGCCA GGTCCCACTC AGGCATACCT TAGGAGGTGG  3661 AAAGTTAGGG TCTGGGCTTC TACCACCATC CCCTGAATTG GGAATATAGT CAGTGGGACA  3721 GTGAGTGAGA AGGAATGGAG CAAGACACAG ATGAATTAGA **GAACTTCCCA CG**CCCCGCCC  3781 AGCCCCTCAC ACTATTGAAG GTCAGCTTGA TGTTGCCTGC GTCCAGTGCG GCAGCCCTCT  3841 TGTCAAAGCT TATGACGTGG GCGAAGTCCT CAAAGGCCGT GTTCACCTCC ACG**CAGAAGC**  3901 **CCC**GGTCCTG TGGGTACAGC AGTGCATGAA GCAGGGGCAC CACGTGCCTG AGGCCAAAGC  3961 TGGCAGGCAG AGT**GGTCACA** TTACAGATGG TCAAGAAGGC CCTTGCCTCT GGAAGAGATC  4021 CACAGGACTG TGGTCTGCAT TAGAAACCAA GTTATCCAAG TCCTCATCAC TTGACAATTA  4081 AGCCCCCATG CCTACCCTTG GTTTGGATGT CACACAGAGC TGAAGGGGTT GGGAGATGTC  4141 TCCCAACTGC TGATTAATGC TGAGTTTCTC AAAATTAACT TTTGTTCCTT TGGCTTAAAG  4201 CTCTGGCCCC AGCTGGGCCC TCCTGCTGCT CAGCAGTGCT TCCATGCATT CTCAATCAAC  4261 A**TTTGCTGG**C CCCTACCCTT TGGGTCTATT TATTGAGTGC TTACTATGTA CCAGGTACCA  4321 TGATGAGTGT TTCACACATT ATCTCATTTA ATCCTCTTGG TTCTACATTT AAGAATAGGT  4381 CCTAGTATCC TAATTTTACA CAGAAGGAGA AACCAAGGTT CAGAGAAACT AGAGCACTTG  4441 CCCAAGGTCT CACAGCTGAT ATACGGCTGG GCAAGAACCC TACCCTGGGG GGGCTCTCTA  4501 TCCCCAAAGT GTCTGCTTTC AAGCACCACA CCCACCCAGA TGACTTAGTC CATGGGAGAA  4561 ACGGAGGCCA CCAGGTGTGC AGCTCCCCAC CAATACTAAA AACATGTCCA CAGGCCAGGC  4621 TCTATGCTTC TCATTTAGGC CTCACAACAT TCCAGTG**AAG CAGAGGCTTA GA**AAAGTGAT  4681 CTCAGTTGGC CCAAGATGAC ATGAGTGGTA AATGAAGCCA CTGGAGTTCA AAGTCTGGCT  4741 CTCACGTCAG TTAAGCACAG GCCAGATTCC CTTTCTCTCC CTCCACATAG CTCTCACCTC  4801 CTCTGGCCTT TTTGCTGATG GCAGAGGTGT GCTCCTCTTT CTTCTTTCCA AGGGTACTTC  4861 CTCAACCCTG CTACAGTCCT TATCAACACA GCATTCATTC TCAGGGAATT GAAAGCTCCA  4921 AGTCACCCTT GTTACTGGCT CCTTCCATCA AGCAGACACC TCACCTAACC TGGAACATCC  4981 TTTCCACCAT GCTCTCCCCT TCATCAAGCT AACTACCTCT GCTCTTTTCC ATTGTTTCAA  5041 AACCTCTTGA AATGCAGCAG AAAGGAAAAG GAATAAACAT TTATTGAGAA CCTATTAGAC  5101 ACTTTACAGA TGTTATTTAA TTCTCACAAC CTTACCTATG AATTATCCCT GATTTACAGG  5161 TGAGCAAACA GGCTCAGAGA CACGGAACTT ACGGAGGTCT CAGAGCCAAT AAGCAACCAG  5221 GAGTAATCTG ACTCTAAAAC ATGTGCTGCC CCCATGCCAC CTCCATGACT CTACTCTCCC  5281 AAAGGTTGCT TGTGTCTACT AGCCAAATCC AAGGCCGCCT TCTTTGTCCT CACTCATCTG  5341 GTCCTCAGTG CCTCTACCAC TTACTTCTTC AACACTCCCC TCTCCA**GGTC ACA**CCCTCTC  5401 TGACCTTTTA TTCTCTTGCA TATGCATGTT CCTGAGAACA GCACTTAGTC CTCTTTCACT  5461 ACCCAATCTG TACTCCCTCG CAATCTCATC CTTCATCTCT CCTGATTTTC AAACATCCTA  5521 GACTGGATAT TTCAGACACT TAAAATTCAA CAGATCTCAA GAGAATATAT TTCTCATGAG  5581 CATCCTCCAT GGATCTCCCT GACCCGGATC TGTCTGCCTG TTCTAAGTCC AGGGCTCTTT  5641 GCATTATCCT ATGGGATGAA AGGCACCATC ATGGTCTGCC TGGCTGTGGA AAGCAAGAGG  5701 GACTCCTGGG CTCATCTGCC CTCCATAGGC AGGAACCCAA ATGGGGAAAG AGGAAGGGAA  5761 CTGTCTAAAA AAGTTGCTTT CTCAGGTTGT GTGGAGAGAC AGGACTCAGT GAGGATAGGC  5821 CCCTACAGGC CTATCAACCA GCCACTCTGT GGCTAAAAGT TAATTGTCCA ATAAAATTTA  5881 GCTATCCCAG GATAAGGCAT TTCTACTTTA TACGATTATT CTGTTTTAAA ATATATTTGC  5941 TTTGAGAGTA ATTCCCCAAA TAGAATTCCT ATTTTCTACA GCCTTGCTCT TCTTGTTTTA  6001 TCCTTATCAA TAGTGCCATC ATCCACTGCC CTGACACCTC TGATTCCTTC CTTTCCCTCC  6061 ATTTCACAAA ATCTATCAAT TCTACTTAGT GACTCTATCC CACTGGTGTT GAATTCTCCC  6121 TTTTTTTTTT AAACAGCAGA AACAGTTTAT TAAAATAGAA ATCTTATGTA GAACCTTTAT  6181 GCATAAAAAA GATTCAAGCT GCTTCACTTG AAAACAGGAA GCAGCACCAA ATCCCCCCAT  6241 ACTCAACT**CC AACCCTTGCT** TGTCTCAAGG CACCTACATA GAAACCCAGA GTGTCTCAGA  6301 AGAACAGCTG GAGCGCTACA GCCTGCTCTC TTCTGCTGTC CCCACTGCCC CCGGCTAGGA  6361 TCACCTCCTA CAAGATTCCT GCACAGCCTA CTGACTTTTC TCCCTGCTCA CCCTCTTCCA  6421 TCCCTCTAAC CCAGCTCCTA GCATCTAGTG CTTCTTGGAC TTTTGTACCA AGTAGCCCCA  6481 ACAGTAGGAG AGAGGTCTGG GGATAAGTGA AATGAATATG ACTATTTGAA TTACCACTAT  6541 CAGTTTTAGT ATCTTGAGGC TTCAAGAACT GCTCTACCTA GCACCACCAC CTTCCCTAAG  6601 GACATAAGTA CGTACTCTAG ACTGAAAGGT TCTACTATAC ACCATCTAAT CTGATCATTC  6661 CCCGGTTCCT CATTACTTAC GAGAGAAAAA TCCAAATTTC TTATTTTAGC ATTCAAGGCA  6721 CTTTACATGC AGGGCTCTAC TTCCAAGCTG GGGCATGTGG TTCCAGAGAA AGAGAGGATA  6781 CCTCAGGACA GTATGATACA TCTTACCTAA TGGTAAGTAA TTTAAAATAT TAAATTTATA  6841 TTAAATAGGT AATATGGTGT TAAAATGCAA CACCTACGTG AATTTGTAAG GCAAAACAGT  6901 ACTTCAAAGA AATGTATATG CAGTGAGACA TTTCTAGCTT TATTTCCAGA TCCCTTTCAA  6961 CCCAGGTACC ATTTAATCTC TGTAATTAGG TGTACTCTGG TGGAACACTC ACCATTTTTT  7021 AGATGAGGAA ACAGGGTCAG AAAGGTCAAG TGACCTATCC AA**GGTCACA**C AACTAGCAAG  7081 CAGTTAAGCT AGACTTTTAC TGTAGGCAGT CTGCCTCCAG TCCCGCTCCT AACCACTGTA  7141 CTGTATTATC TCCTCAAATG CATTAGAATC TTCACTTCCA TAAAGAAATA TGCTCTTTGT  7201 TTTTTTCTTT TGGAACACAA GGTTTAGTCC ATCACTCACT CACTTTTCCA CTTTTGATAA  7261 GATGTAAATA TTGAGAACTA TTTCTCTTCT CCAGTAGAAG **AAGAGCAACA GGCC**ATGTGT  7321 GATGGTAAAT GGCAGCTGAC ACTTGCGACA GTATCAAGAA GGCACTAAGG AATGGTGAGA  7381 AGGATGACTT GCCCTTTCTA AAGTGGCATC TCAGCTTGAG TCTGCAGAAT GCCTTGCAAA  7441 TCCAGCTCAG TACTGCTAGA TAATCCATAT TGACAAGAGA AGCTAAAGAT TCAGATTTTC  7501 TATGTGAAGT TTCCTGGTTT TAAAATGTTA GCAACAAATT CCAAATTTTT ACAGAACACT  7561 GTACAGGCCA GAAAGAACAA TCTGTGTGCT ACCAGTTTAC AATTAGCTAT GCTTCTTCCT  7621 CCTCAGACCT GGCA**TGCCCT TT**CCACCCCC TTGCCTTTGC TCTTAGTACC CCGTGTACAC  7681 TCCAACCTCT TCCACCTACA AATTTCTAAA TGCACAGTTC AAATGTTAGT TCTTCAGATT  7741 CCTGTCTCTG TAATAACACT TGCCCCTTTG TATAACAGTT CTCATGTATA TCTTCCTATT  7801 AGACCATGAG CTCCTGTGGT AGAAATTGTG TATTCATTAC TGTATCCCTA GTGTGCAGTA   1. GATCACA GTAG \| \|  1. *PXDN* 3’UTR sequence with bioinformatically predicted hsa-miR-7973 target sequences.   microT CDS v5.0 predicted target sequence locations are marked in green: positions 12-31 and  329-354. TargetScan and miRDB commonly predicted seed sequence location is marked in red:  position 2298-2305. \| \| --- \| --- \| --- \| --- \| \| 1 GCTCCTGGGA G**GCTCCTCAG AGTTTGTCTG C**TGTGCCATC GTGAGATCGG GTGGCCGATG  61 GCAGGGAGCT GCGGACTGCA GACCAGGAAA CACCCAGAAC TCGTGACATT TCATGACAAC  121 GTCCAGCTGG TGCTGTTACA GAAGGCAGTG CAGGAGGCTT CCAACCAGAG CATCTGCGGA  181 GAAGGAGGCA CAGCAGGTGC CTGAAGGGAA GCAGGCAGGA GTCCTAGCTT CACGTTAGAC  241 TTCTCAGGTT TTTATTTAAT TCTTTTAAAA TGAAAAATTG GTGCTACTAT TAAATTGCAC  301 AGTTGAATCA TTTAGGCGCC TAAATTGAT**T TTGCCTCCCA ACACCATTTC TTTTT**AAATA  361 AAGCAGGATA CCTCTATATG TCAGCCTTGC CTTGTTCAGA TGCCAGGAGC CGGCAGACCT  421 GTCACCCGCA GGTGGGGTGA GTCTTGGAGC TGCCAGAGGG GCTCACCGAA ATCGGGGTTC  481 CATCACAAGC TATGTTTAAA AAGAAAATTG GTGTTTGGCA AACGGAACAG AACCTTTGAT  541 GAGAGCGTTC ACAGGGACAC TGTCTGGGGG TGCAGTGCAA GCCCCCGGCC TCTTCCCTGG  601 GAACCTCTGA ACTCCTCCTT CCTCTGGGCT CTCTGTAACA TTTCACCACA CGTCAGCATC  661 TAATCCCAAG ACAAACATTC CCGCTGCTCG AAGCAGCTGT ATAGCCTGTG ACTCTCCGTG  721 TGTCAGCTCC TTCCACACCT GATTAGAACA TTCATAAGCC ACATTTAGAA ACAGGTTTGC  781 TTTCAGCTGT CACTTGCACA CATACTGCCT AGTTGTGAAC CAAATGTGAA AAAACCTCCT  841 TCATCCCATT GTGTATCTGA TACCTGCCGA GGGCCAAGGG TGTGTGTTGA CAACGCCGCT  901 CCCAGCCGGC CCTGGTTGCG TCCACGTCCT GAACAAGAGC CGCTTCCGGA TGGCTCTTCC  961 CAAGGGAGGA GGAGCTCAAG TGTCGGGAAC TGTCTAACTT CAGGTTGTGT GAGTGCGTTA  1021 AAAAAAAAAA AAAAAAAGAA TCCCTATACC TCATTTGTAT TTTTAAAATG CGTGATGTTT  1081 TATGAAATTG TGTCCATTTT TTAGGTATTA GATATGGCAG AAAAACCATT TCCACTATGC  1141 AAAGTTCTTT TAGACGTCAG TGAAAATCAA CTCTCATACC TCATGGTCTC TCTTTAATTG  1201 ACCAAAACCT TCCATTTTTC TCTAAATACA AAGCGATCTG TGTTCTGAGC AACCTTTCCC  1261 CGAACACACA GCTTCAGTGC AGCACGCTGA CCTGAGTATC CACCATGTGC CAGGCACAGT  1321 GCTGGGCACA CGAGGCACCA AGGTCCGGGC CACCTGCCCG CAGCAAGGCC CAGCTGAGGT  1381 GGTGGAGGGA GCCCCTGAGG TCAGGGGCCG TTTCGGTTCA GGGTGGCAGG TGTCCAGCAC  1441 TGGGGTATGG CGTCGAGGCT TCCATGGGGT GGGGGAGGCC AGCTTCCTTC TGACAGGATG  1501 GGCGCATACA GTGCCTGGTG TGATTTGTGC ACAACCCGTG TTCCAGGTGC ACATCCTCCC  1561 AAGGAGACAC CCAGACCCTT CCAGCACGGG CCGGCCAAGT TGCTGCGGCG GAGGCAGCAT  1621 TTCAGCTGTG AGGAAGGTCA TTGGATTCAT GTGTTTTATC TGTAAAAATG GTTGTCTTAA  1681 CTTCTTAACC TCATATTGGT AAGTGATTGA TAAAAATTGG TTGGTGTTTC ATGACATGTG  1741 GACTTCTTTT GAAATAGCAA GTCAAATGTA GTGACCAAAT TGTGGAAGAG ATTTCTGTCA  1801 AATAGGAAAT GTGTAAGTTC GTCTAAAAGC TGATGGTTAT GTAAGTTGCT CAGGCACTCA  1861 GATGACAGCA GATTCTGGGT TCTGGGAGTG TTCTGTGCCT CTTACATGCC CTGGAGGCCT  1921 CATGGTCTCA GTGCTGAGGC GGCACACCTG TAGCACACCT GCGTAATGTG CGGTCTGGGC  1981 CAGTCACAAG GAATTGTGTT GTCTAAGCCA AAGGGGGAAG CTGACTGTGA TTTACCAAAA  2041 AAAATTCTGT AATTCAAACC AAAATGTCTG CGGAATCACC AGTTTGATAC TCTCTGTAAT  2101 CAGAACAGTG GGCAGTGCCT GGGTGAACGT GTCTAGCAGC CACTGTGCGG GATCGCTGTA  2161 ACAGGAGTGG AATGTACATA TTTATTTACT TTTCTAACTG CTCCAACAGC CAAATGCCTT  2221 TTTTATGACC ATTGTATTCA GTTCATTACC AAAGAAATGT TTGCACTTTG TAATGATGCC  2281 TTTCAGTTCA AATAAAT**GGG TCAC**ATTTTC AAATGGA \| | | |
| --- | --- | --- | --- | --- | --- | --- | --- |
|  | | |
|  |  |  |

| 1. *TGFBR2* 3’UTR sequence with bioinformatically predicted target sequences for hsa-miR548ba and hsa-miR7973. TargetScan and miRDB commonly predicted target seed sequence location for hsa-miR-7973 at position 107-113 is marked in red. miRDB predicted seed sequence location for hsa-miR-548ba at position 810-816 is marked in yellow. TargetScan predicted seed sequence location for hsa-miR-548ba at position 1470-1476 is marked in blue. |
| --- |
| 1 CTCTTCTGGG GCAGGCTGGG CCATGTCCAA AGAGGCTGCC CCTCTCACCA AAGAACAGAG  61 GCAGCAGGAA GCTGCCCCTG AACTGATGCT TCCTGGAAAA CCAAGG**GGGT** **CAC**TCCCCTC  121 CCTGTAAGCT GTGGGGATAA GCAGAAACAA CAGCAGCAGG GAGTGGGTGA CATAGAGCAT  181 TCTATGCCTT TGACATTGTC ATAGGATAAG CTGTGTTAGC ACTTCCTCAG GAAATGAGAT  241 TGATTTTTAC AATAGCCAAT AACATTTGCA CTTTATTAAT GCCTGTATAT AAATATGAAT  301 AGCTATGTTT TATATATATA TATATATATC TATATATGTC TATAGCTCTA TATATATAGC  361 CATACCTTGA AAAGAGACAA GGAAAAACAT CAAATATTCC CAGGAAATTG GTTTTATTGG  421 AGAACTCCAG AACCAAGCAG AGAAGGAAGG GACCCATGAC AGCATTAGCA TTTGACAATC  481 ACACATGCAG TGGTTCTCTG ACTGTAAAAC AGTGAACTTT GCATGAGGAA AGAGGCTCCA  541 TGTCTCACAG CCAGCTATGA CCACATTGCA CTTGCTTTTG CAAAATAATC ATTCCCTGCC  601 TAGCACTTCT CTTCTGGCCA TGGAACTAAG TACAGTGGCA CTGTTTGAGG ACCAGTGTTC  661 CCGGGGTTCC TGTGTGCCCT TATTTCTCCT GGACTTTTCA TTTAAGCTCC AAGCCCCAAA  721 TCTGGGGGGC TAGTTTAGAA ACTCTCCCTC AACCTAGTTT AGAAACTCTA CCCCATCTTT  781 AATACCTTGA ATGTTTTGAA CCCCACTTT**T TACCTT**CATG GGTTGCAGAA AAATCAGAAC  841 AGATGTCCCC ATCCATGCGA TTGCCCCACC ATCTACTAAT GAAAAATTGT TCTTTTTTTC  901 ATCTTTCCCC TGCACTTATG TTACTATTCT CTGCTCCCAG CCTTCATCCT TTTCTAAAAA  961 GGAGCAAATT CTCACTCTAG GCTTTATCGT GTTTACTTTT TCATTACACT TGACTTGATT  1021 TTCTAGTTTT CTATACAAAC ACCAATGGGT TCCATCTTTC TGGGCTCCTG ATTGCTCAAG  1081 CACAGTTTGG CCTGATGAAG AGGATTTCAA CTACACAATA CTATCATTGT CAGGACTATG  1141 ACCTCAGGCA CTCTAAACAT ATGTTTTGTT TGGTCAGCAC AGCGTTTCAA AAAGTGAAGC  1201 CACTTTATAA ATATTTGGAG ATTTTGCAGG AAAATCTGGA TCCCCAGGTA AGGATAGCAG  1261 ATGGTTTTCA GTTATCTCCA GTCCACGTTC ACAAAATGTG AAGGTGTGGA GACACTTACA  1321 AAGCTGCCTC ACTTCTCACT GTAAACATTA GCTCTTTCCA CTGCCTACCT GGACCCCAGT  1381 CTAGGAATTA AATCTGCACC TAACCAAGGT CCCTTGTAAG AAATGTCCAT TCAAGCAGTC  1441 ATTCTCTGGG TATATAATAT GATTTTGAC**T ACCTTA**TCTG GTGTTAAGAT TTGAAGTTGG  1501 CCTTTTATTG GACTAAAGGG GAACTCCTTT AAGGGTCTCA GTTAGCCCAA GTTTCTTTTG  1561 CTTATATGTT AATAGTTTTA CCCTCTGCAT TGGAGAGAGG AGTGCTTTAC TCCAAGAAGC  1621 TTTCCTCATG GTTACCGTTC TCTCCATCAT GCCAGCCTTC TCAACCTTTG CAGAAATTAC  1681 TAGAGAGGAT TTGAATGTGG GACACAAAGG TCCCATTTGC AGTTAGAAAA TTTGTGTCCA  1741 CAAGGACAAG AACAAAGTAT GAGCTTTAAA ACTCCATAGG AAACTTGTTA ATCAACAAAG  1801 AAGTGTTAAT GCTGCAAGTA ATCTCTTTTT TAAAACTTTT TGAAGCTACT TATTTTCAGC  1861 CAAATAGGAA TATTAGAGAG GGACTGGTAG TGAGAATATC AGCTCTGTTT GGATGGTGGA  1921 AGGTCTCATT TTATTGAGAT TTTTAAGATA CATGCAAAGG TTTGGAAATA GAACCTCTAG  1981 GCACCCTCCT CAGTGTGGGT GGGCTGAGAG TTAAAGACAG TGTGGCTGCA GTAGCATAGA  2041 GGCGCCTAGA AATTCCACTT GCACCGTAGG GCATGCTGAT ACCATCCCAA TAGCTGTTGC  2101 CCATTGACCT CTAGTGGTGA GTTTCTAGAA TACTGGTCCA TTCATGAGAT ATTCAAGATT  2161 CAAGAGTATT CTCACTTCTG GGTTATCAGC ATAAACTGGA ATGTAGTGTC AGAGGATACT  2221 GTGGCTTGTT TTGTTTATGT TTTTTTTTCT TATTCAAGAA AAAAGACCAA GGAATAACAT  2281 TCTGTAGTTC CTAAAAATAC TGACTTTTTT CACTACTATA CATAAAGGGA AAGTTTTATT  2341 CTTTTATGGA ACACTTCAGC TGTACTCATG TATTAAAATA GGAATGTGAA TGCTATATAC  2401 TCTTTTTATA TCAAAAGTCT CAAGCACTTA TTTTTATTCT ATGCATTGTT TGTCTTTTAC  2461 ATAAATAAAA TGTTTATTAG ATTGAATAAA GCAAAATACT CAGGTGAGCA TCCTGCCTCC  2521 TGTTCCCATT CCTAGTAGCT AAA |
