# Supplementary material for: Target prediction and validation of microRNAs expressed from FSHR and aromatase genes in human ovarian granulosa cells
Source: Sci Rep. 2020 Feb 10;10:2300. doi: 10.1038/s41598-020-59186-x (PMC7010774; doi:10.1038/s41598-020-59186-x)
Supplement: Supplementary file 2 — Supplementary Information2. [file 41598_2020_59186_MOESM2_ESM.docx]

**Target prediction and validation of microRNAs expressed from FSHR and aromatase genes in human granulosa cells**

Ilmatar Rooda^1,2^, Kati Hensen^3^, Birgitta Kaselt^1^, Sergo Kasvandik^4^, Martin Pook^3^, Ants Kurg^3^, Andres Salumets^2,5,6,7^, Agne Velthut-Meikas^1,2,*^

^1^Department of Chemistry and Biotechnology, Tallinn University of Technology, Tallinn, Estonia

^2^Competence Centre on Health Technologies, Tartu, Estonia

^3^Institute of Molecular and Cell Biology, University of Tartu, Tartu, Estonia

^4^Proteomics Core Facility, Institute of Technology, University of Tartu, Tartu, Estonia

^5^Institute of Clinical Medicine, Department of Obstetrics and Gynecology, University of Tartu, Tartu, Estonia

^6^Institute of Biomedicine and Translational Medicine, Department of Biomedicine, University of Tartu, Tartu, Estonia

^7^Department of Obstetrics and Gynecology, University of Helsinki and Helsinki University Hospital, Helsinki, Finland

**Supplementary Methods**

**Small RNA RT-qPCR data analysis – selection of endogenous control for normalization**

Eight miRNAs and U6 snRNA in 16 primary granulosa cell samples (8 cumulus and 8 mural granulosa samples) were tested for the stability of their expression levels. Additionally, artificial small RNA spike-in was added to these samples in order to estimate the pipetting error. All primers and the spike-in were purchased from Exiqon. The results are displayed in the table below. Except for the artificial spike-in, hsa-miR-132-3p demonstrated the lowest variability between samples.

| miRNA | Average Ct |  | SD |
| --- | --- | --- | --- |
| hsa-miR-126-3p | 25.92664 |  | 1.804009 |
| hsa-miR-129-2-3p | 31.4689 |  | 3.058987 |
| hsa-miR-129-5p | 31.46952 |  | 1.952898 |
| **hsa-miR-132-3p** | **22.26479** |  | **0.757571** |
| hsa-miR-142-5p | 31.15986 |  | 2.684086 |
| hsa-miR-196a-5p | 30.10833 |  | 1.822812 |
| hsa-miR-223-3p | 23.63032 |  | 2.244144 |
| hsa-miR-30a-5p | 27.03187 |  | 2.457037 |
| hsa-miR-874 | 27.61752 |  | 1.138007 |
| Artificial Spike-in | 20.52509 |  | 0.340899 |
| U6snRNA | 19.98738 |  | 2.323012 |

The selection of the 8 miRNAs presented in the table was based on the small RNA sequencing results of primary human granulosa cells ^1^.

**Proteomics sample preparation**

Cells were suspended in 10 volumes of 4% SDS, 100 mM Tris-HCl pH 7.5, 100 mM DTT lysis buffer. Samples were heated at 95^o^C for 5 min, followed by probe sonication (Bandelin) (60x 1 s pulses, 50% intensity). Unlysed material were cleared with centrifugation at 14 000 g for 10 min, although, lysis was noted to be nearly complete. 30 µg of protein was precipitated with 2:1:3 (v/v/v) methanol:chloroform:water. Protein pellets were suspended in 25 µl of 7 M urea / 2 M thiourea 100 mM ABC solution, followed by disulfide reduction and cysteine alkylation with 5 mM DTT and 10 mM chloroacetamide for 30 min each at room temperature. Proteins were predigested with 1:50 (enzyme to protein) Lys-C (Wako Chemicals) for 4 h, diluted 5 times with 100 mM ABC and further digested with trypsin (Sigma Aldrich) overnight at room temperature. Peptides were desalted with in-house made C18 StageTips. For determining suitable peptides for targeted MS, a single digested lysate was further fractionated with a basic reversed phase fractionation (bRP) into 7 fractions using a step-wise elution with the following buffers:

| Buffer A | 200 mM AF pH 10 |
| --- | --- |
| Buffer B | 200 mM AF pH 10, 10% ACN |
| Buffer C | 200 mM AF pH 10, 15% ACN |
| Buffer D | 200 mM AF pH 10, 17.5% ACN |
| Buffer E | 200 mM AF pH 10, 20% ACN |
| Buffer F | 200 mM AF pH 10, 30% ACN |
| Buffer G | 200 mM AF pH 10, 80% ACN |

**LC/MS/MS analysis**

Peptides were injected to an Ultimate 3000 RSLCnano system (Dionex) using a 0.3 × 5 mm trap-column (5 µm C18 particles, Dionex) and an in-house packed (3 µm C18, Dr Maisch) analytical 50 cm × 75 µm emitter-column (New Objective).

For peptides fractionated with bRP, each fraction was analysed with an A to B 8-40% 120 min gradient (buffer A: 0.1% FA, buffer B: 80% ACN + 0.1% FA) and eluted at 200 nl/min to a Q Exactive Plus (Thermo Fisher Scientific) MS/MS using a nano-electrospray source (positive mode, spray voltage of 2.6 kV). The instrument was operated with a top 10 data-dependent acquisition (DDA) strategy by measuring a 350-1400 m/z MS (at a resolution setting of 70 000) scan and then triggering fragmentation for the ten most intense ions (resolution setting of 17 500). Dynamic exclusion was set to 40 s and normalized collision energy (NCE) of 26 was used.

For peptides used for targeted analysis, elution was carried out at 250 nl/min with an A to B 10-45% 60 min gradient. The MS was operated in a scheduled parallel reaction monitoring (PRM) mode by isolating and fragmenting only peptides from the selected proteins within ±4 minute windows of their retention times. Retention time scheduling was calibrated using the indexed retention time (iRT) method ^2^. MS/MS isolation window was 1.0 m/z and scans were performed at a resolution setting of 17 500. Ion target value of 2e5 and fill time of 60 ms were used. NCE was set to 26.

**LC/MS/MS data analysis**

For the discovery data, MS raw files were processed with the MaxQuant software package (1.4.0.8) ^3^. Methionine oxidation, and protein N-terminal acetylation were set as variable modifications, while cysteine carbamidomethylation was defined as a fixed modification. Search was performed against UniProt (www.uniprot.org) human reference proteome database (downloaded: 2015 October) using the tryptic digestion rule (cleavages after lysine and arginine without proline restriction). Only identifications with at least 1 peptide ≥ 7 amino acids long (with up to 2 missed cleavages) were accepted and transfer of identifications between runs based on accurate mass and retention time was enabled. Peptide-spectrum match and protein false discovery rate (FDR) was kept below 1% using a target-decoy approach ^4^. All other parameters were default.

Targeted MS raw files were analysed with the Skyline software ^5^. Spectral library was created from Mascot (Matrix Science) database search results derived from measuring synthetic peptides (JPT Technologies) with data-dependent LC/MS/MS mode. Peptides with the highest intensity in the library were preferred for the targeted analysis. Peptides with methionines and cysteines were excluded. Only y-ions (starting from ion 4, y4 up to last ion -1) with charge states +1 and +2 were allowed. All extracted ion chromatogram (XIC) integrations were manually inspected for correct peak picking. Fragment XIC traces with strong interference and erroneously picked peaks (mass errors > ±20 ppm, lack of fragment coelution) were removed.

**References**

1. Velthut-Meikas, A. *et al.* Research resource: small RNA-seq of human granulosa cells reveals miRNAs in FSHR and aromatase genes. *Mol. Endocrinol. Baltim. Md* **27**, 1128–1141 (2013).

2. Escher, C. *et al.* Using iRT, a normalized retention time for more targeted measurement of peptides. *Proteomics* **12**, 1111–1121 (2012).

3. Cox, J. & Mann, M. MaxQuant enables high peptide identification rates, individualized p.p.b.-range mass accuracies and proteome-wide protein quantification. *Nat. Biotechnol.* **26**, 1367–1372 (2008).

4. Elias, J. E. & Gygi, S. P. Target-decoy search strategy for increased confidence in large-scale protein identifications by mass spectrometry. *Nat. Methods* **4**, 207–214 (2007).

5. MacLean, B. *et al.* Skyline: an open source document editor for creating and analyzing targeted proteomics experiments. *Bioinforma. Oxf. Engl.* **26**, 966–968 (2010).
