# Supplementary material for: Target prediction and validation of microRNAs expressed from FSHR and aromatase genes in human ovarian granulosa cells
Source: Sci Rep. 2020 Feb 10;10:2300. doi: 10.1038/s41598-020-59186-x (PMC7010774; doi:10.1038/s41598-020-59186-x)
Supplement: Supplementary file 3 — Supplementary Information3. [file 41598_2020_59186_MOESM3_ESM.docx]

**Target prediction and validation of microRNAs expressed from FSHR and aromatase genes in human granulosa cells**

Ilmatar Rooda^1,2^, Kati Hensen^3^, Birgitta Kaselt^1^, Sergo Kasvandik^4^, Martin Pook^3^, Ants Kurg^3^, Andres Salumets^2,5,6,7^, Agne Velthut-Meikas^1,2^

^1^Department of Chemistry and Biotechnology, Tallinn University of Technology, Tallinn, Estonia

^2^Competence Centre on Health Technologies, Tartu, Estonia

^3^Institute of Molecular and Cell Biology, University of Tartu, Tartu, Estonia

^4^Proteomics Core Facility, Institute of Technology, University of Tartu, Tartu, Estonia

^5^Institute of Clinical Medicine, Department of Obstetrics and Gynaecology, University of Tartu, Tartu, Estonia

^6^Institute of Biomedicine and Translational Medicine, Department of Biomedicine, University of Tartu, Tartu, Estonia

^7^Department of Obstetrics and Gynecology, University of Helsinki and Helsinki University Hospital, Helsinki, Finland

**Supplementary Results**


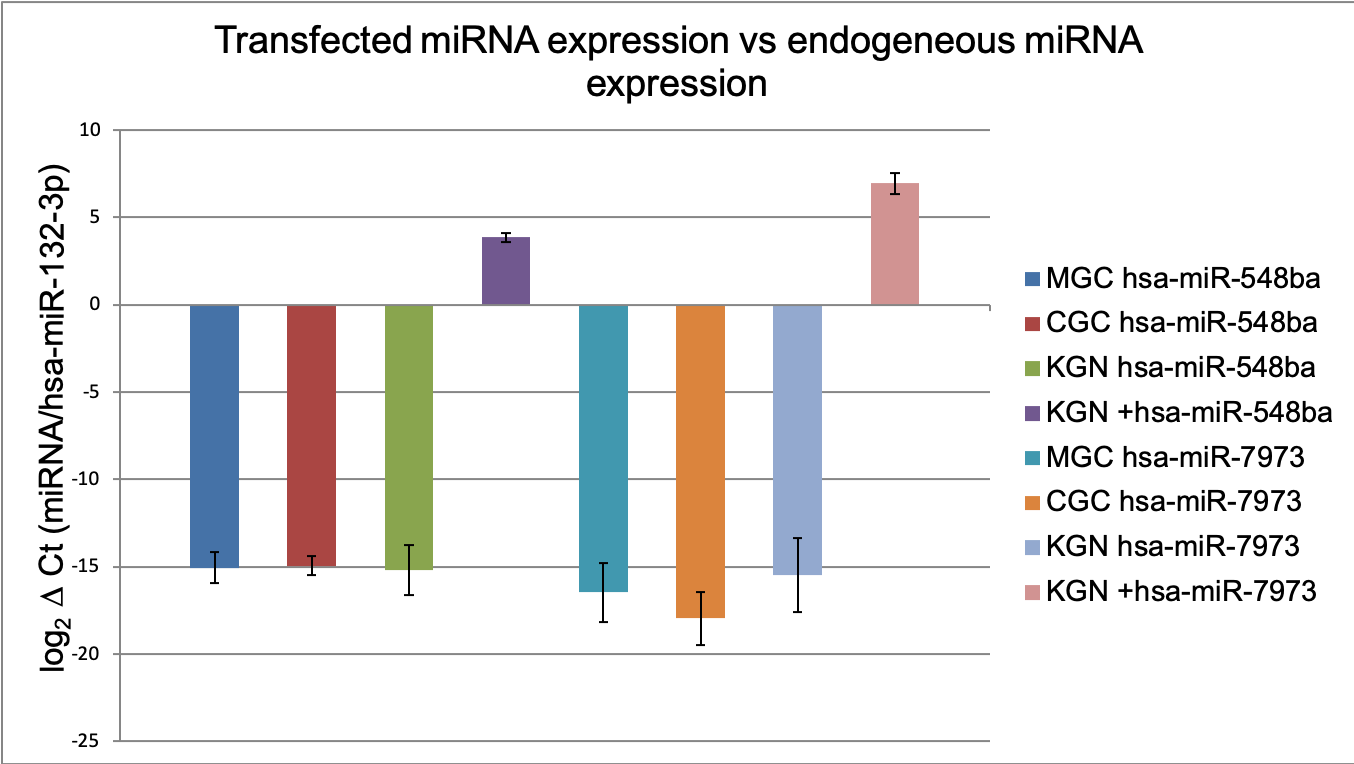


**Supplementary Figure 1.** Transient hsa-miR-548ba and hsa-miR-7973 expression in KGN cell-line compared to primary human granulosa cells. For positive control human mural (MGC) and cumulus granulosa cell (CGC) RNA was used (n=8). miRNA expression in KGN cells is shown as average expression of four parallel samples ±SD on log_2_ scale. miRNA expression levels were normalized against miRNA hsa-miR-132-3p levels. Transfected KGN samples are indicated with + sign in the legend (yellow and pink colour).


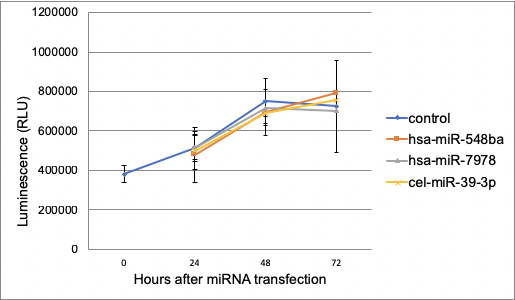


**Supplementary Figure 2.** KGN cell viability upon transient miRNA expression. Luminescence signal was measured from cells transfected with hsa-miR-548ba, hsa-miR-7973 or control miRNA cel-miR-39-3p at three time-points: 24, 48 and 72 hours. Signal from non-transfected cells (control) were measured at 0, 24, 48 and 72 hours. The indicated value refers to normalized luminescence: signal from all lysed cells minus signal from dead cells. Results are shown as average of 3 independent experiment with ± SD. Student t-test p-values were above 0.05, no significance difference in living cell number were detected between non-transfected control samples and transfected cells.


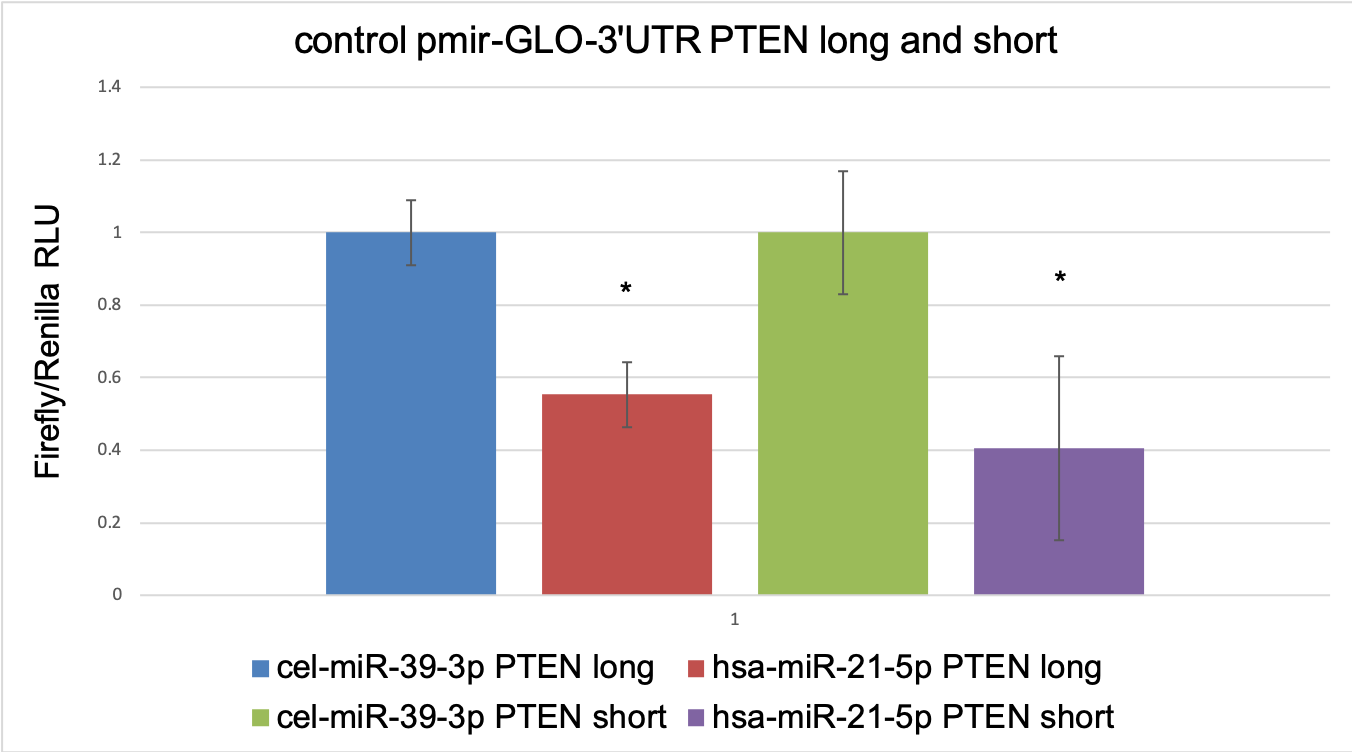


**Supplementary Figure 3.** Suppression of luciferase signal upon hsa-miR-21-5p binding to PTEN 3’UTR. pmirGLO-3’UTR-PTEN vector and hsa-miR-21-5p were used as a positive control for miRNA:mRNA binding to confirm the luciferase assay reliability. Two versions of PTEN 3’UTRs were used: pmirGLO-3’UTR-PTEN long represents 3’UTR length obtained from UCSC genome browser and pmirGLO-3’UTR-PTEN short represents miRDB miRNA target prediction program version of 3’UTR.Results are shown as average of 3 independent experiment with ± SD (*p < 0.05; Student t-test).


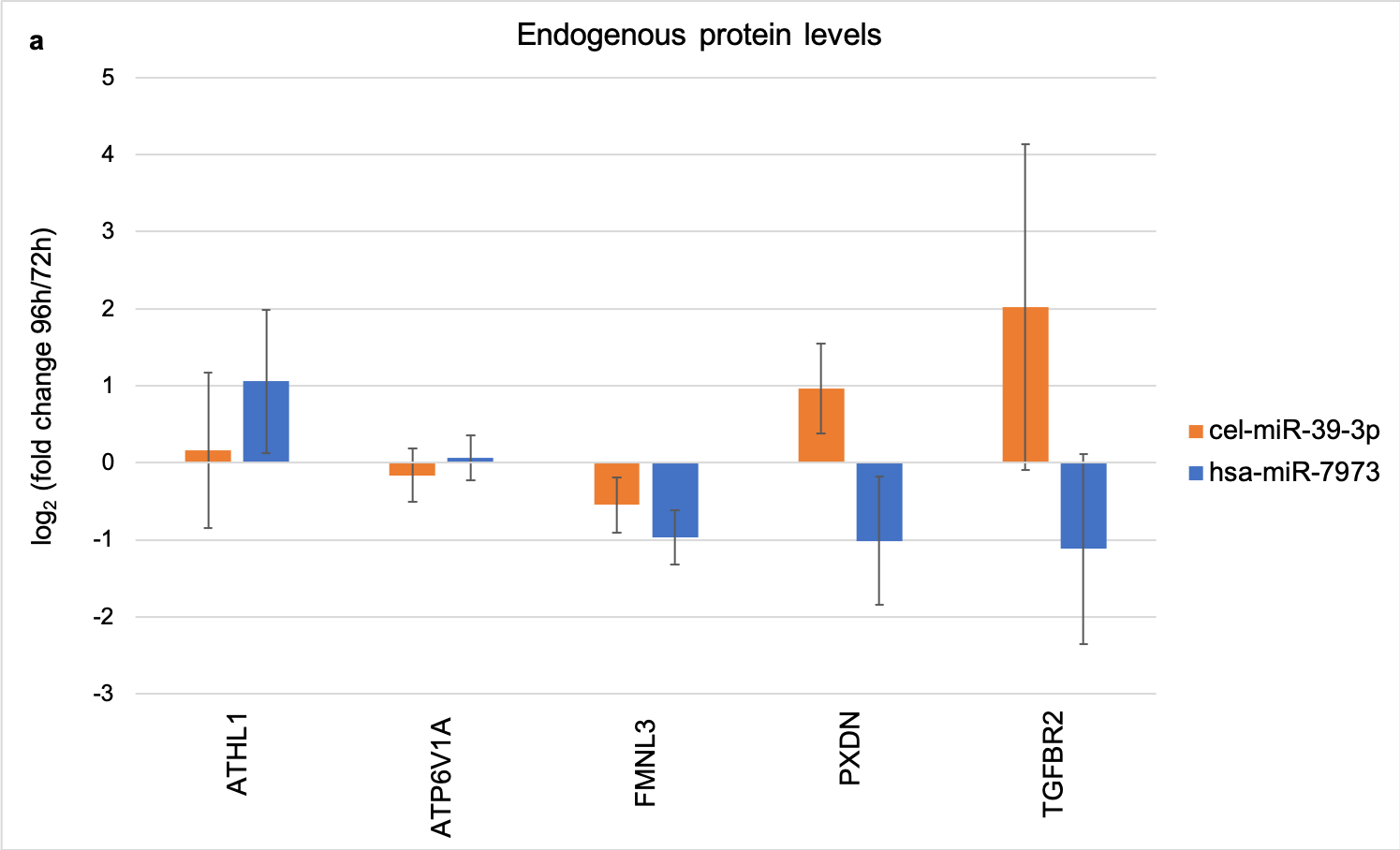


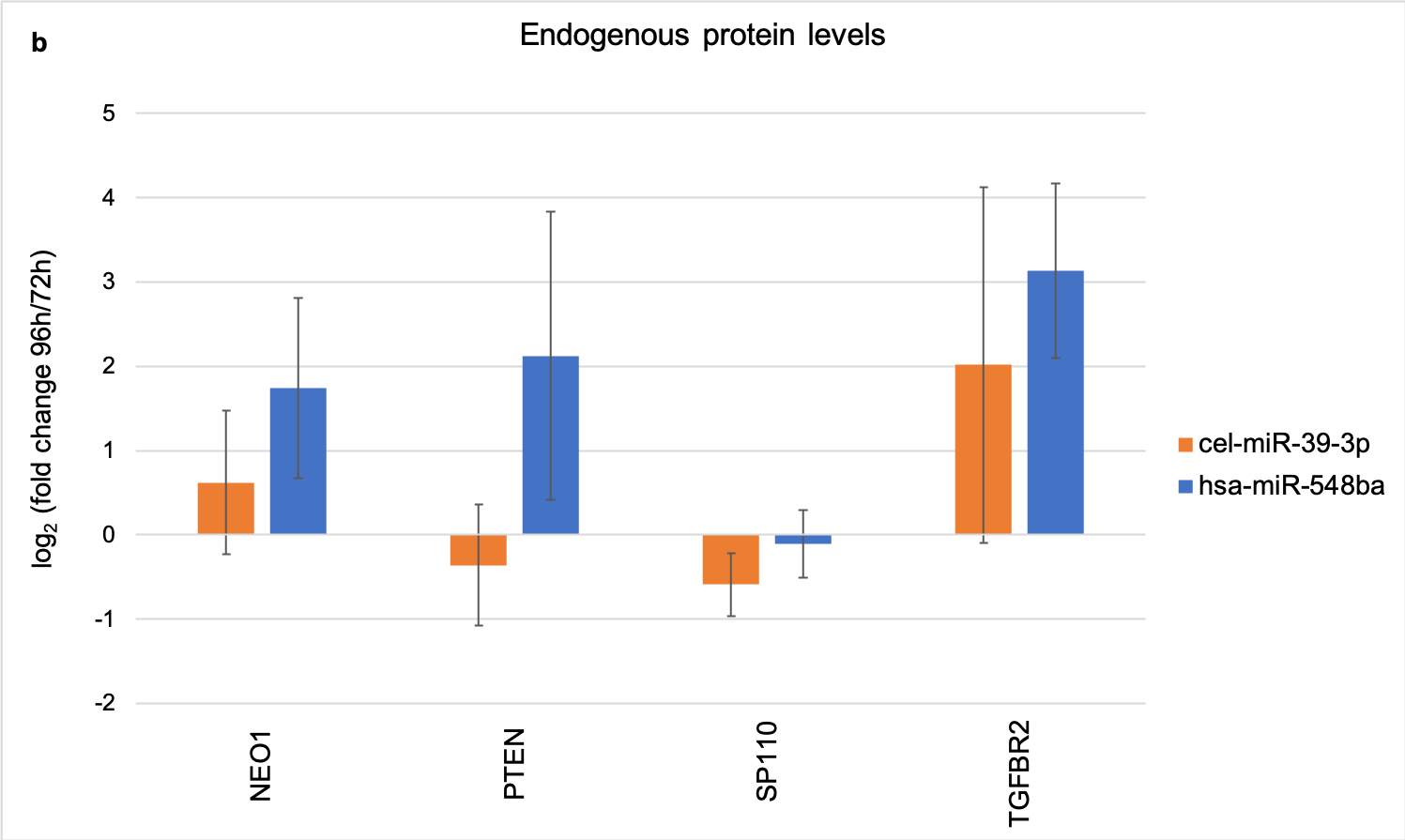


**Supplementary Figure 4.** Endogenous expression levels of target proteins upon KGN cell line transfection with hsa-miR-548ba (A) or hsa-miR-7973 (b) mimics. Protein levels were assessed at two time-points (72h and 96h) after miRNA transfection and normalized against GAPDH. Results are displayed as average fold change between time-points ±SEM on log_2_ scale, n=3.

**
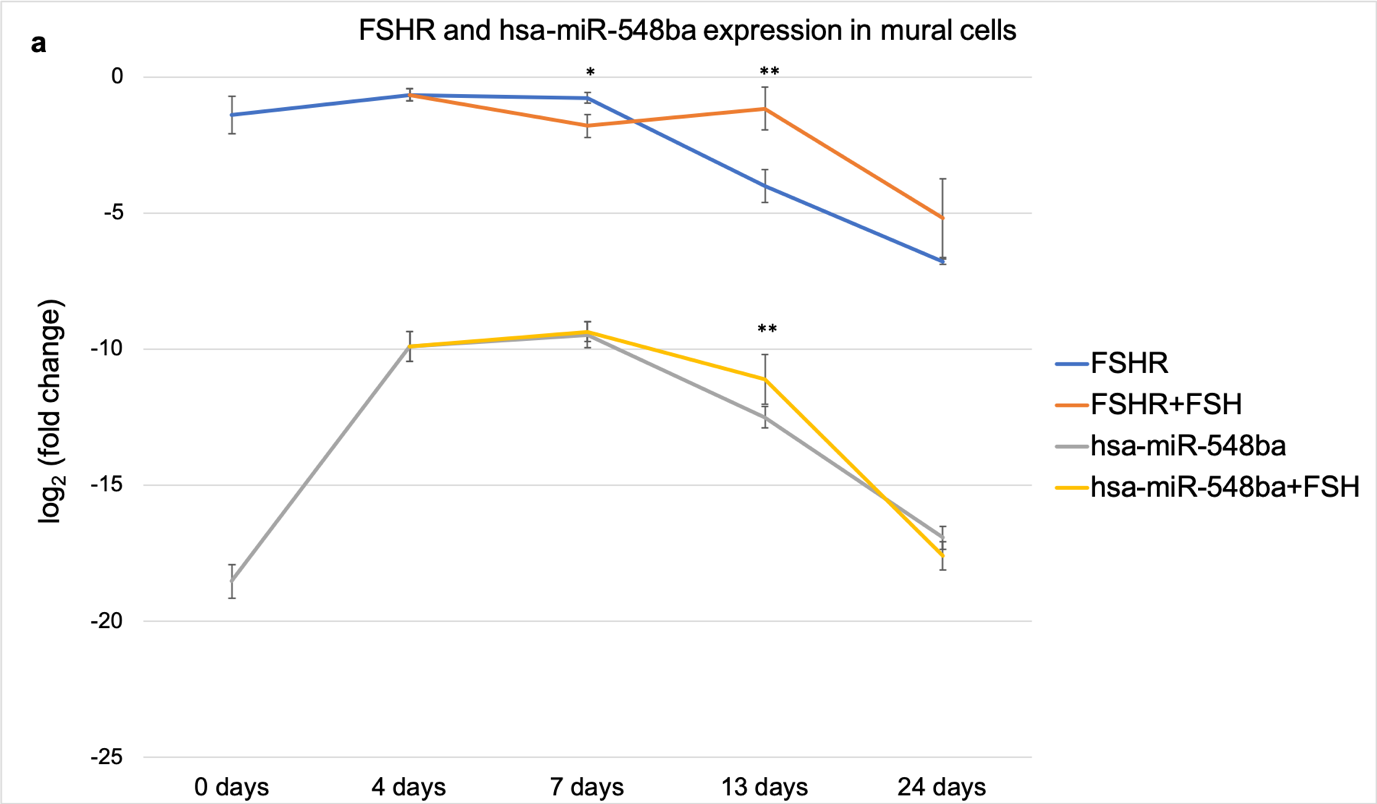
**

**
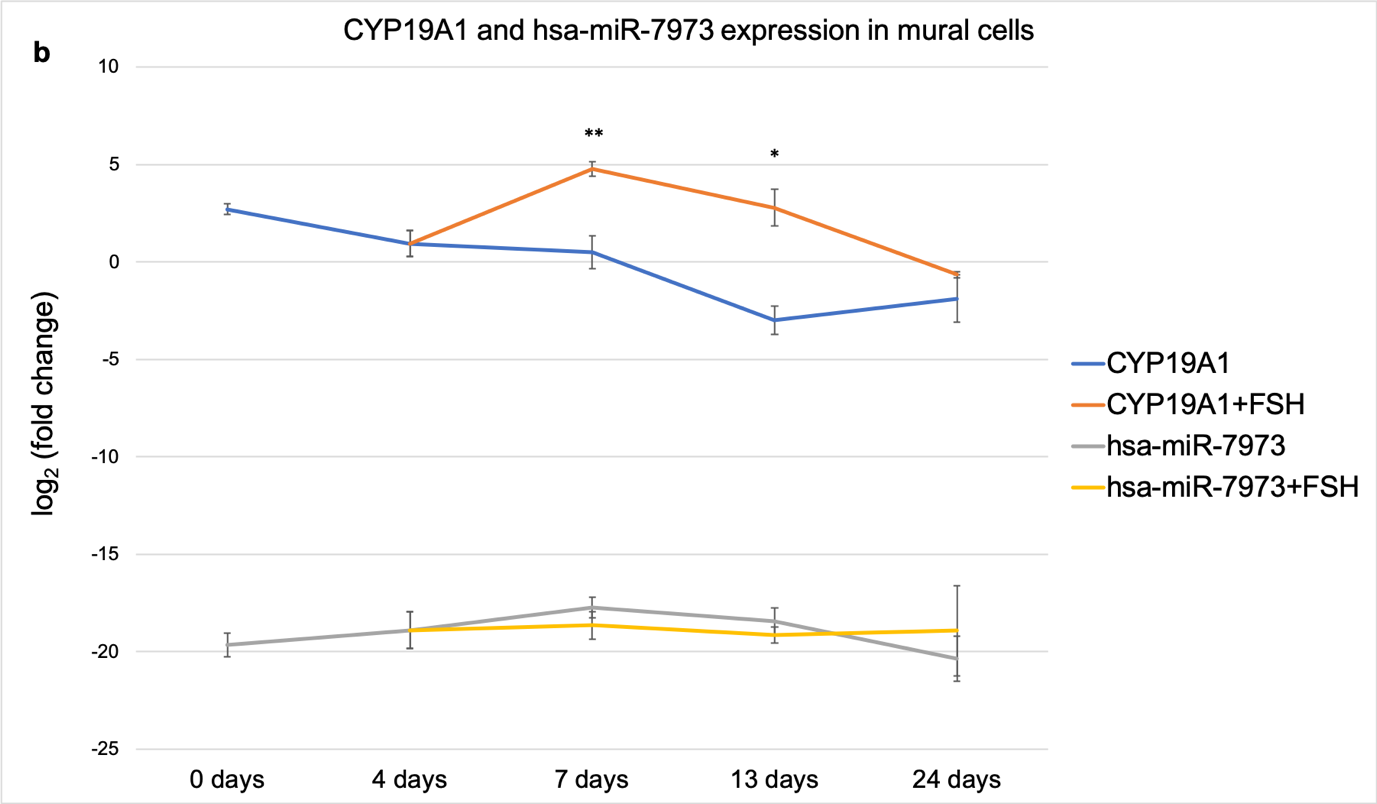
**

**Supplementary Figure 5.** miRNA and host gene expression levels in primary human mural granulosa cells. mRNA and miRNA expression levels were normalized to SDHA or hsa-mir-132-3p, respectively. Results are displayed as average fold change ±SD on log2 scale, n=3, (*p < 0.05; **p < 0.01; Student t-test between cells exposed to 1 IU/ml FSH vs non-treated cells). A) hsa-miR-548ba and FSHR expression and B) hsa-miR-7973 and CYP19A1 expression
